# Expression Variation: Its Relevance to Emergence of Chronic Disease and to Therapy

**DOI:** 10.1371/journal.pone.0005921

**Published:** 2009-06-15

**Authors:** Anatoly L. Mayburd

**Affiliations:** CPA Global, Alexandria, Virginia, United States of America; University of Glasgow, United Kingdom

## Abstract

**Background:**

Stochastic fluctuations in the protein turnover underlie the random emergence of neural precursor cells from initially homogenous cell population. If stochastic alteration of the levels in signal transduction networks is sufficient to spontaneously alter a phenotype, can it cause a sporadic chronic disease as well – including cancer?

**Methods:**

Expression in >80 disease-free tissue environments was measured using Affymetrix microarray platform comprising 54675 probe-sets. Steps were taken to suppress the technical noise inherent to microarray experiment. Next, the integrated expression and expression variability data were aligned with the mechanistic data covering major human chronic diseases.

**Results:**

Measured as class average, variability of expression of disease associated genes measured in health was higher than variability of random genes for all chronic pathologies. Anti-cancer FDA approved targets were displaying much higher variability as a class compared to random genes. Same held for magnitude of gene expression. The genes known to participate in multiple chronic disorders demonstrated the highest variability. Disease-related gene categories displayed on average more intricate regulation of biological function vs random reference, were enriched in adaptive and transient functions as well as positive feedback relationships.

**Conclusions:**

A possible causative link can be suggested between normal (healthy) state gene expression variation and inception of major human pathologies, including cancer. Study of variability profiles may lead to novel diagnostic methods, therapies and better drug target prioritization. The results of the study suggest the need to advance personalized therapy development.

## Introduction

The studies of gene expression variability started relatively lately, with the advent of high-throughput technologies of analysis [1], [2]. These studies revealed a striking and fundamental fact that despite identical genotypes, individuals within the same species at the same conditions express gene products at very different levels. These quantitative differences span a range of several orders of magnitude [3]. In a recent large scale study both extrinsic and intrinsic character of such variations was shown [4]. Since “health” status can be defined as homeostatic balance, the ability of fluctuations to propagate along regulatory chain is related to the ability to induce dramatically different cellular states based on bi-state/bi-stability model [5]. The effect of expression stochasticity upon spontaneous differentiation of progenitor cells was studied in [6]. According to the publication, stochastic fluctuations in the turnover of two proteins, Notch and Delta, might underlie the random emergence of neural precursor cells from initially homogenous cell population. If stochastic alteration of the levels in signal transduction networks is sufficient to spontaneously alter a phenotype, can it cause a sporadic chronic disease as well?

A study was published comparing non-disease and disease state, detecting de-regulation, as a signature of disease mechanism [7]. Another study points to the link between excessive expression of non-mutated protein in chromosomal trisomy and the risk of Alzheimer's disease development in the age of fifties for the affected individuals [8]. The publication proceeds to extrapolate this observation to the general causes of neurodegenerative disease. The review [8] also discusses the impact of non-mutated gene expression upon the probability of sporadic prion disease, taupathies, Parkinson and Alzheimer's disease. Many earlier publications also present the connection between anomalous gene dosage and development of neurodegenerative disease [9]. Such situation qualitatively differs from variations of gene expression at normal gene dosage, making the work [8] especially important, since it appears to produce such an interpretation of variation vs. disease. The publications [10]–[13] consider stochastic origin of diseases including tumors in the condition of haploinsufficiency. In such cases a single gene copy does not produce enough of a transcription factor (tumor suppressor) to always ensure a concentration above the critical [10]–[12]. Since the function of stochastically modulated signal transducer can be up-stream, the effect of such fluctuations is exceedingly leveraged [13].

While providing a link between expression variability and disease, the prior publications appear to be confined to particular diseases (neurodegenerative, particular tumors) and certain genes, thus they do not provide a global view of the possible connection between normal gene expression variance and mechanism of subsequent sporadic disease emergence. By contrast, this work presents a genomic scale study into all major chronic diseases, including aging and such a scope may be of interest.

## Results

### Elevated expression variability associates with disease

**Figure 1:**
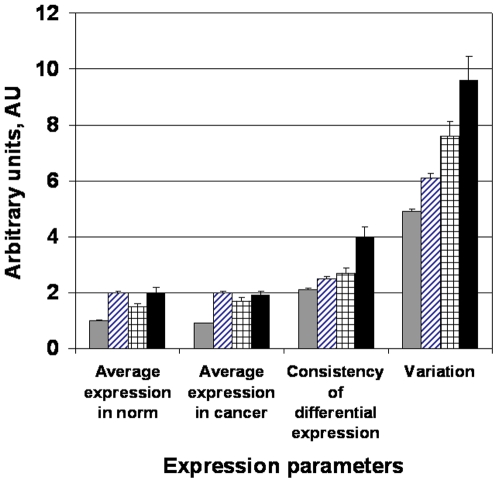
Expression parameters of random genes vs the parameters of therapeutic anti-cancer targets. Presented is a comparison of expression parameters for random genes (grey bars), cancer-related genes, both target and non-target (striped bars), proposed and developing anti-cancer targets (checkered bars) and successful anti-cancer targets (black bars). The parameters of expression were estimated as described in the Methods. The differential expression refers to the comparison between norm and cancer. The confidence intervals were computed with the significance level α = 0.05

Figure 1 presents normalized levels of expression, consistency of differential expression and integrated panel variability for 54675 probe-sets comprising the high density U133 Plus 2.0 microarray platform by Affymetrix. For cancer-related genes (∼2900 probe-sets) variability is higher in norm as compared to random genes. The same refers to differential expression and expression. For prospective anti-cancer targets, the expression parameters correlate with the extent of clinical development, being higher for FDA approved targets (black bars) as compared with the mix of target and non-targets (striped bars, “cancer-related” category).

Since variability was measured in normal state, its link to the propensity of a gene to become a successful target is significant. Typically, participation in the essential mechanism of pathogenesis establishes a gene as a target. On the other hand, the elevated expression variation was measured prior to development of a disease – hence it may be causative to the subsequent pathogenesis. A concern exists that the extent of clinical development may not objectively reflect the extent of mechanistic participation of a gene but may be distorted by other factors, such as market niche, the historical duration of study, dominant opinions in the field etc. To ensure that the extent of variability indeed parallels the objective extent of mechanistic participation, the variability data were aligned with differential expression consistency and metric of tissue-specific expression. Prior works show that differential expression consistency is an objective metric providing significant enrichment in the FDA-approved and proposed anti-cancer targets [21]. Such a link provides indirect measure of relevance to the disease mechanism. The criterion of tissue-specific expression is another routine computational filter [22] in target selection and is independent vs. non-mechanistic (marketing) factors. Ideally, it seeks the target candidates over-expressed only in a particular lineage and absent in all the rest. Thus both systemic and the lineage-related side-effects are minimized.

**Figure 2 pone-0005921-g002:**
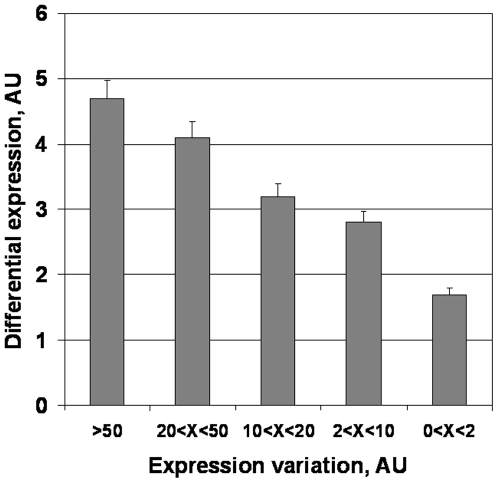
Differential expression as an alternative criterion of mechanistic involvement vs expression variation. Differential expression consistency in norm vs cancer transition was aligned with variability levels. Total population of random and disease-associated genes was included in the analysis.

Figure 2 illustrates a link between consistency of differential expression in transition from norm to cancer and expression variability in the norm. According to the Figure 2, the increased tendency to be differentially expressed in cancer is directly proportional to variability in normal state.

**Figure 3 pone-0005921-g003:**
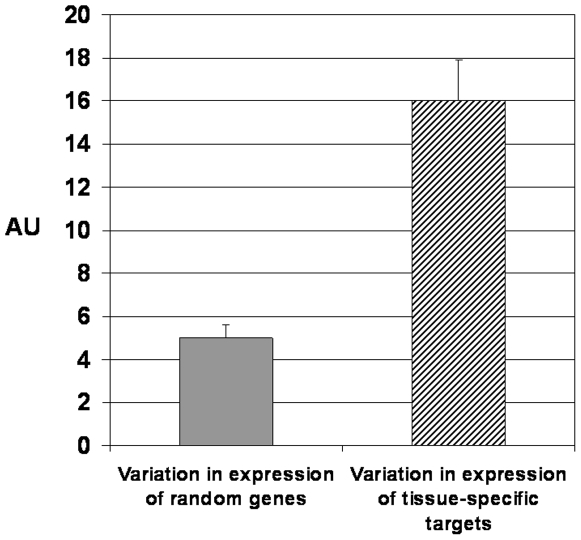
Comparative expression variation of random genes and tissue-specific anti-cancer targets. The variability level in the random gene class (grey bar) was compared with tissue-specific anti-cancer target candidates and targets (striped bar). The latter sub-set was formed by selecting genes expressed in a single tissue lineage and over-expressed in cancer.

Figure 3 compares random genes and the populations of prospective and approved anti-cancer targets selected by the criteria of tissue-specific expression, see Methods and more detailed presentation in Supporting materials (Text S1, pages 29–46). In the group of ∼190 probe-sets simultaneously top ranked by MAXc/AV, MAXc/MAX_N_, MAXc/VULNERABLES the level of variability in the norm was by far the highest. At the same time, this group of genes was strongly enriched in FDA-approved targets and proposed target candidates, such as MAGE (A3, A6, A2, A11), MS4A1, REG4, MSLN, IL1A, ENPEP, TYR, RARA, FCLRA. The data by Figure 2 and 3 provide an additional link between anti-cancer target enrichment and variability, thus leading to propose variability's mechanistic role.

**Figure 4 pone-0005921-g004:**
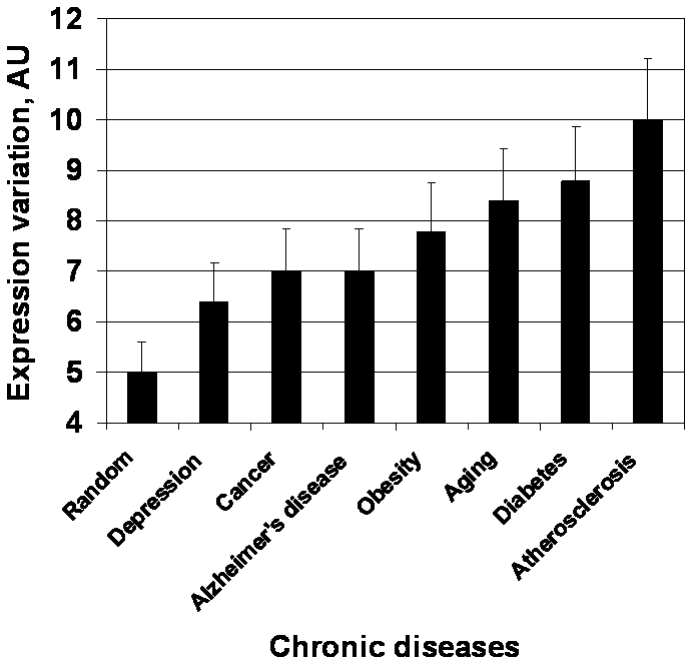
Expression variability in chronic diseases. Averaged panel variability of gene expression was measured for different chronic disease states, including aging (250–500 probes-sets per a disease).

To ensure that these observations are not specific for cancer alone, similar analysis was conducted for other classes of disease-related genes, see Figure 4. Mining the database “Genes” at NCBI with keywords corresponding to particular disorders produced gene aliases associated with these disorders based on the analysis of scientific and medical literature. The expression variability trend first discovered for anti-cancer targets vs random genes was confirmed for all major chronic conditions.

**Figure 5 pone-0005921-g005:**
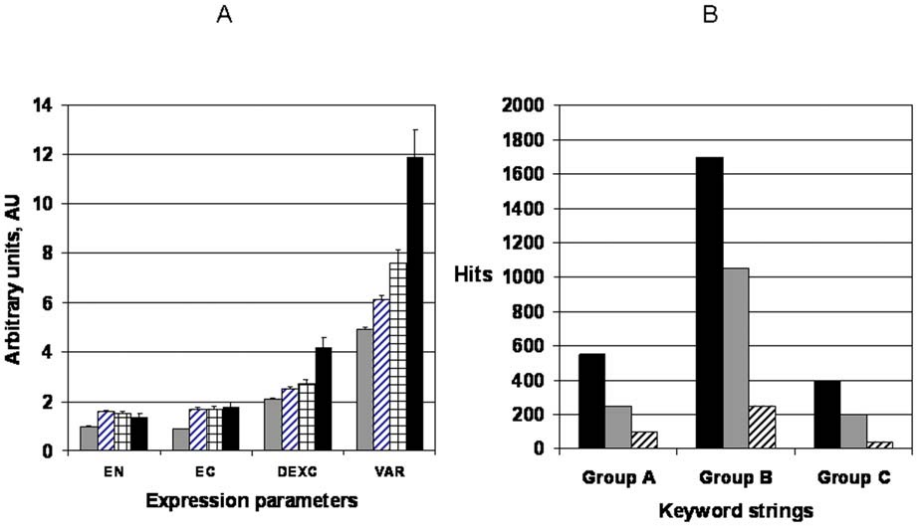
Variation of gene's expression correlates with the gene's association with chronic disease. A. Relationship between participation in multiple chronic conditions and parameters of expression. Striped bars – the genes participate in 0 chronic conditions, Checkered bars – participation in 1 chronic conditions, grey bars – participate in 2–3 chronic conditions, black bars - participates in 4–7 chronic conditions. The parameters of expression comprise EN – expression in norm; EC – expression in cancer, DEXC – differential expression consistency between norm vs. cancer; VAR – variability of expression. B. In this computational experiment, three categories of gene expression were identified: highly-variable (black bars), at average variation level (grey bars) and at minimal variation level (striped bars). The lists of genes of equal size (1000) were selected to be a part of Boolean query of the structure: Group A: [(gene list) and ((biomarker* or (diagnostic adj marker*) or (prognostic adj marker*))]. Group B: [(gene list) and (disease or disorder)]. Group C: [(gene list) and (longevity or mortality)]. Each gene list of differing variability was incorporated in the query of a patent database (Micropatent by Thomson) and the numbers of hits were plotted for each group, designated as above.

Figure 5 addresses a baffling phenomenon of a gene's multiple participation in several chronic conditions, most notably the similarity between the set of genes active in neurodegenerative disease and cancer [16]. Other multiple participation parallels were observed, such as between obesity and depression [17], [18]. While the latter link can be also explained by behavioral and psychological changes, an alternative explanation calls for a common signal pathway involvement [19], [20]. The lists of gene aliases (extracted as described in Methods) were aligned and the Index of Multiple Participation was computed. According to the data, each gene participated in ∼2 chronic disorders on average and thus our findings support the prior isolated observations that genes active in the mechanism of a single disorder may be a part of multiple disease mechanisms [16]–[20]. The expression parameters of such multiple participants were plotted in Figure 5.A. The degree of gene expression in norm (EN) and cancer (EC) was increasing for disease participants vs. random genes. Even more prominent trend was observed for differential expression consistency (DEXCON) and variability (VAR) that were steadily increasing in proportion to the Index of Multiple participation, being maximal for multiple participants.

Figure 5.B presents the results of querying of a patent database with Boolean keyword strings, comprising a combination of a gene list and terms describing disease association (P3.2). Under comparable conditions, the gene list selected from the highest variability category produced 4–8 fold greater number of hits as compared to the gene list of the same size selected from the least variable category. Figure 5.B points to a strong correlation existing between the level of expression variability and the extent of disease association.

### Validation of results

A possibility exists that the differences between random control and disease-associated genes are not objective, but arise accidentally due to a particular composition of the integrated panel. To rule this possibility out, multiple (8) sub-panel compositions were generated by random bootstrapping and in each composition random genes were compared with therapeutic target genes (P3.3). The difference between the groups under comparison exceeds the relevant confidence intervals.

**Figure 6 pone-0005921-g006:**
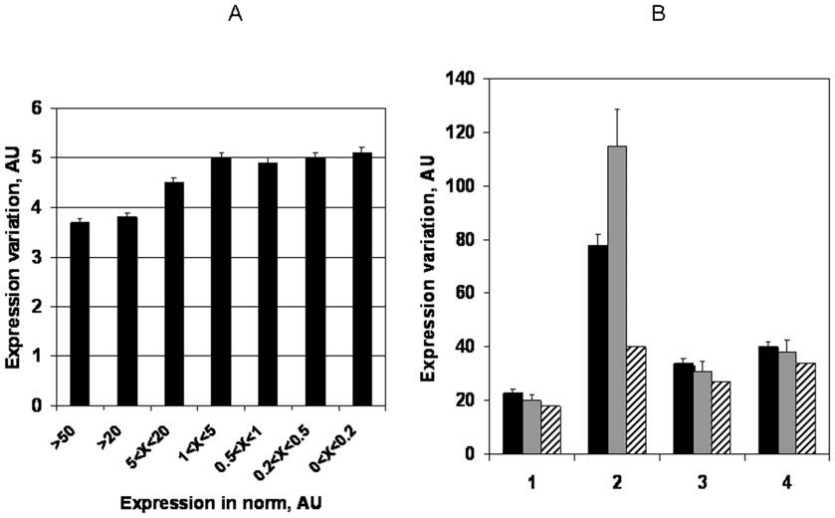
Validation of results. A. Expression variability measured as a function of transcript copy-number. The 54675 expression values measured on a single microarray chip were normalized, with the average copy-number set to 1. The normalized values were split into brackets, expressed in the arbitrary copy-number units. B. Comparison of different expression variability metrics. The groups 1 and 2 represent MAX/MIN; the groups 3 and 4 represent Coefficient of Variation (CVx50), brought to the comparable scale. The groups 1 and 3 belong to variations measured in normal tissues, while the groups 2 and 4 refer to cancer tissues. The black bars stand for the targets of FDA approved anti-cancer drugs, the grey bars stand for the developing and proposed anti-cancer targets, striped bars indicate random genes.

A hypothesis was advanced that the elevated variability in disease-related categories may be a function of higher expression also observed in these categories. To test this possibility, variability as a function of copy-number was plotted (Figure 6.A) using multiple brackets of copy number values (in arbitrary units). The observed relationship pointed to higher variation at lower copy-numbers, running counter to the above mentioned hypothesis.

Still another possibility of an artifact resides in the fact that a very sensitive measure was employed as a variation criterion. A propagated error, associated with a ratio of outliers can be very significant and an additional test is needed to evaluate its neutralization by aggregation of multiple datasets in a panel. Coefficient of Variation (CV) was chosen as a less sensitive, but more reliable alternative metric, taking into account the scattering behavior of the entire population of N values in a project. Figure 6.B presents comparative variability for random genes and disease-associated categories.

The Figure 6.B indicates that the trend, observed using MAX/MIN is preserved while using CV (compare groups 1 and 3). High confidence interval for the group 2 still allows confirmation of the same trend in comparison with the group 4. Of note, variability and especially measured by MAX/MIN is significantly greater in cancer tissues, reflecting expression deregulation.

### Quantitative ontological analysis

Per processing as discussed in Methods, total gene population formed ∼7500 functional categories, with 3900 of those having non-zero population. Functional enrichments between the variability class and the total gene pool were computed (see folder P4 of Supporting online materials). The high variability class displayed maximal enrichments for the genes controlling tissue and organ development, proliferation, muscle contraction, chemotaxis, ion channel functioning, neurotransmitter release and processing, immune response. By contrast, low variability class displayed enrichments for the genes controlling enzymatic metabolic reactions, structural proteins, cell division, ribosome, translation factors. The analysis of variability extremes was followed by the study of individual diseases (Supporting materials, section P4). The results indicate that the most-enriched functional categories correspond to the currently accepted disease mechanism. For example, tissue morphogenesis and proliferation regulation was the dominant category for cancer, neurotransmission – for depression, etc. This result suggests that disease-associated variability is concentrated among mechanistically essential genes.

**Figure 7:**
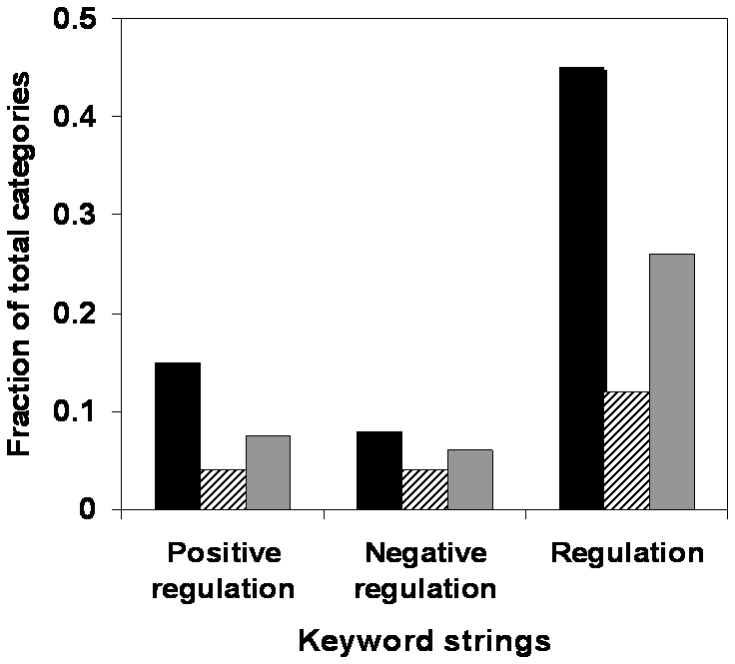
Functional analysis of gene categories displaying opposing extremes of expression variability. Comparative results of key-word searching of the most and least disease-related functional categories. The 7500 functional categories produced by AMIGO ontological classification were filtered resulting in ∼3900 with non-zero population. Multiple randomly drawn sets of genes (500-1000 in size) served as negative control. The functional enrichment coefficients (FENR) were computed in the AMIGO-represented negative control and similarly treated disease-related datasets. The strings of FENR formed random and disease-related sub-profiles in each functional category. The sub-profiles were compared by T-test and p-values were sorted. The functional categories with the least p-values (best 10% of rank, p<10–11) were termed “most disease-related” (black bars). The functional categories with the highest p-values (>0.9) were termed “least disease-related” (striped bars). Grey bars stand for the total population of AMIGO-derived functions. The most and the least disease-related groups of functional categories were searched using the keyword combinations, such as “regulation”, “positive regulation” and “negative regulation”. The fractions of the functions responding to the keyword combinations were computed and plotted.

The FENR values were assembled in the panel, with two sub-profiles in every populated functional category, one for random negative control and another for the diseases being grouped together. Such grouping allowed exploration of the features generic to all chronic disorders using the rationale presented in Methods. The functional categories ranked based on p-value of T-test vs. random negative control were subjected to text-mining, as well as the total list of categories. The results are given by Figure 7.

According to the plotting, top-ranking disease-related functional categories respond to the keyword “regulation” twice as frequently as the total population of categories and four fold more frequently if compared with lowest-ranking categories (representing random gene population). More surprising, however, was the finding that positive regulation is much more prominent among top-ranking disease-related categories as compared to the lowest ranking categories or total list of categories. The total population of categories appears to display approximate balance between positive and negative regulation, according to our analysis. This balance between positive and negative regulation appears to shift in favor of positive regulation in the categories most associated with disease and this observation suggests some fundamental biological role.

**Figure 8:**
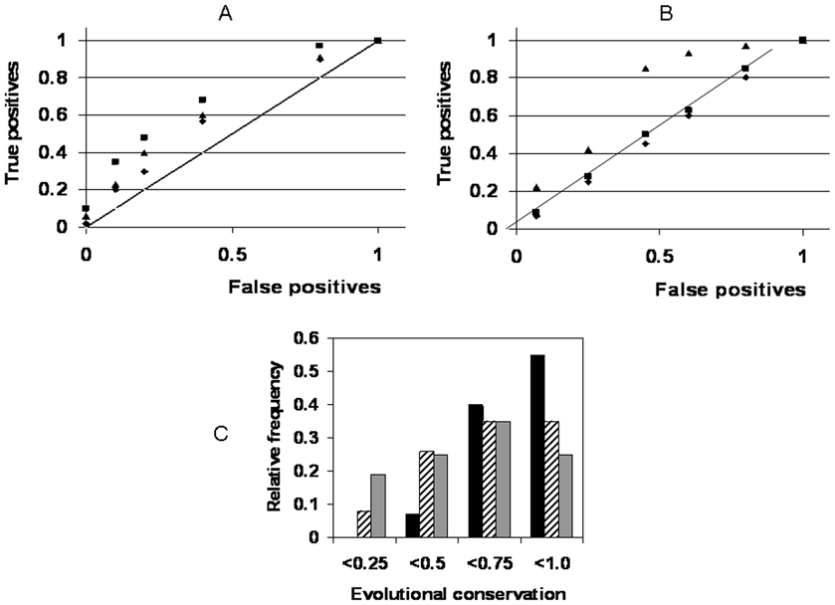
Analysis of pharmacological target efficiency in the context of expression variability. A. Resolution of successful anti-cancer targets (black squares), developing anti-cancer targets (black triangles), non-cancer disease targets (black diamonds) as a function of a combined variation score. The score is obtained by integrating MAX/MIN, coefficient of variation, kurtosis and differential expression criteria of expression variation. The genes were ranked by the combined score in the descending order and at each fraction of the rank the fractions of random genes and the corresponding targets were computed. The results are presented as ROC curves. B. Resolution of successful antibiotic targets, developing targets and random genes as a function of variation. The ROC curve presents “false positives” – random population (black diamonds) ranked in ascending order by MAX/MIN of transcription profile, 0 corresponding to the minimal MAX/MIN, 1 corresponding to the maximal. “True positives” refer to the targets of successful antibacterial drugs (black triangles) and developing antibiotic targets (black squares). The integrated numbers of true and false positives, reached at a particular fraction of the ranking were counted, converted into fractions and plotted vs. fraction of the ranking. C. Distribution of successful antibiotic targets (black bars), developing antibiotic targets (striped bars) and random genes (grey bars) as a function of evolutional conservation. The conservation is defined by a fraction of organisms in a phylogenetic profile expressing the orthologs of the given target. The profile comprises 26 bacterial species according to Cluster Of Ortholog Groups database (COG at http://www.ncbi.nlm.nih.gov/COG/, 2004 edition).

Practical applications of the current project were explored below. Figure 8.A compares the targets of FDA approved anti-cancer drugs, proposed and developing anti-cancer targets, targets of non-cancer disease therapies and random genes, plotted as a ROC curve as a function of ranked variability score. The said score is a combined variation metric, comprised of individual features of MAX/MIN, CV, kurtosis and differential expression consistency. In the context of Figures 1–4, it follows that anti-cancer successful targets display higher variability than the corresponding candidate genes and the magnitude of variability may (to a point!) be a predictor of clinical success. In fact, the odds of high-variation vs. low variation gene to be a target of anti-cancer drug or si-RNA approach differ ∼15–20 fold on the opposing edges of a ranked dataset. The odds ratio reaches 5 fold, comparing average variability and high variability candidates.

The trend observed for non-cancer targets appears to be the opposite: the candidate genes (Figure 4) appear to be somewhat more variable than the finally approved targets (Figure 8.A), while the latter are somewhat more variable than random genes.

Figure 8.B presents the result of the study of *E.coli* gene expression (Supporting Online Materials Text S1, p. 23–24; folder P6). Different features, exploring expression variation (same as in Figure 8.A) were applied to random genes and pharmacological targets. Increased proportion of un-annotated genes was observed in high-variation subset.

On the contrary, the population of successful antibiotic targets displayed decreased expression variation, while displaying increased evolutional conservation (defined as an ability to be expressed in a profile comprised of selected species, Figure 8.C).

## Discussion

### A. The link between expression variation and disease: putative novel diagnostic tests

The main result of the current research is in the finding that expression variability of disease-associated genes is higher than that of random genes. Several possibilities of an artifact were considered (see validation section of Results) and found to be absent. Assuming validity of these findings, the above trend was observed in healthy state and a causal link to inception of pathology may be hypothesized (hypothesis 1). An alternative (hypothesis 2) calls for elevated variability to be a hallmark of a gene's disease-relatedness, but no direct role in pathological mechanism can be attributed.

The references 5, 6, 8, 10–12, 21 support the hypothesis 1, pointing to a possibility of disease inception due to dramatic positive or negative variation in expression of a single gene. Indeed, decrease in a single gene expression level due to haplo-insufficiency of tumor suppressors is carcinogenic. Conversely, engineered over-expression of a signal protein triggers cancer in normal skin [21]. Apparently, similar outcomes can follow anomalous expression due to stochastic variation of gene expression level. Such variations may arise at pre-natal stage. Epigenetic factors and accompanying stochastic noise may exert fateful influence at the stage of zygote. At this point only a few transcript copies are available per a locus and the disproportionate consequences of random fluctuations may define systemic expression profile [5]. Considering rapid onset of differentiation in zygote, this profile may get permanently imprinted, exerting an impact on future health, disease and longevity status of an individual [22]. By this or by a combination of conceivable mechanisms, the resulting cellular population becomes very heterogeneous in terms of systemic expression profiles [23]. According to the hypothesis 1, a fraction of each population is essentially pre-pathological due to insufficient or excessive gene expression. The selective evolutional pressure (and likely existence of controlling processes) requires the fraction of this borderline sub-population to be small at least toward the end of reproductive age. However, the systemic resilience appears to decrease after a certain point in age, as a consequence the weight of the mal-functional cellular population and the severity of this malfunction increases with age. Thus, the link between disease and expression variability can be qualitatively explained. If such interpretation is correct, it suggests a potential application of variation measurements as diagnostic tests for disease predisposition.

Indeed, increased cell-to-cell variation of a given gene expression would indicate loose regulatory control and the possibility that the given gene expression may reach extreme values, high or low. Such extreme values are argued in this report to be the “flashpoints” of sporadic disease. In some types of pathology (cancer, sporadic prionic disease) only a single “extreme” cell may be sufficient to cause systemic effect. In other pathologies (Alzheimer's disease, atherosclerosis, stroke, heart attack) a critical weight of deregulated population has to be reached. In all cases, increased variability in expression levels would facilitate reaching of the critical “triggering” parameters, thus its direct measurement may have diagnostic and prognostic value. A combination of instabilities in regulation of expression of several mechanistically important genes may exert effects comparable to mutations. Currently, the classifiers of disease etiology and prognosis utilize SNP, microarray, proteomic and metabolomic data aligning particular pattern signatures with clinical correlates [24], [25]. A recent trend is to use peripheral blood samples for such purposes [26], [27]. Mostly, the current methods rely on point mutations (RFLP markers, SNP, polymorphisms). In this report we propose measuring cell-to-cell variations of gene expression in the peripheral blood sample taken from a given individual.

Variability studies performed over single monocytes of a peripheral blood sample from the given patient and covering the most informative high-variability gene subset may bring an additional dimension to genetic marker methodology. Thus, variametric component should enhance genetic polymorphism analysis regarding predisposition to chronic disease, diagnostics of congenial disorders, longevity, personalized diet and therapy. On the technical side, multiple expression levels in single cells comprising a sample can be measured by the novel methods of single cell arrays, phosphocytometry and cellomics [28], [29].

### B. The expression variation study and its relation to the progress of anti-cancer therapy

Our data facilitate understanding of limitations existing in cancer therapy and also suggest novel therapeutic possibilities. For example, we demonstrate (Figures 1, 3, 8) that pharmacological targets display increased expression variability.

The effect is especially striking for tissue-specific anti-cancer targets that are in the current focus of research relying on differential expression (Figure 6).

We have conducted a genomic-scale differential expression study. The latter comprised 40 pairs of normal versus cancer Affymetrix array datasets, covering most of tissue environments in a single computational space. The goal was to observe cancer-specific hyper-expression absent in the entire panel of norm. The initial hypothesis stated that such hyper-expression would be a reliable basis for high quality target candidates. Preferably, such hyper-expression should have been tissue-specific. Surprisingly, we observed that such target candidates display the highest variability in all categories studied in this report (Figure 3). Based on this finding, situations are possible when personalized expression profile of a target dramatically differs from the population average profile. The genes over-expressed in cancer vs norm at population level and used as selective targets may be down-regulated at the level of an individual. Conversely, the genes mediating the side effects can be over-expressed in normal tissues and be silenced in a tumor. Such combinations of expression parameters are very likely to cause failure of therapy. In this case extreme cancer target variability would play against the patient. However, opposite situations are possible, when the therapeutic target is extremely over-expressed in tumor, while the side effect determinants in the normal tissues are rudimental. Such situations may lead to increased chance of success. The fact that cancer expression is poorly regulated is trivial. However, the fact that this “regulation defect” is especially concentrated in the subset of genes, proposed for anti-cancer therapy is very meaningful.

We show (based on reliability theory) that the survival probability would be impacted by these fluctuation factors in the most dramatic manner. Consequently, we emphasize **personalized** target visualization approaches taking into account increased target variability described in this report. Some genes – such as metalloprotease MMP12 – display very favorable variation profile, being almost uniformly over-expressed in cancer and almost absent in norm. Conjugating visualizing and therapeutic moieties to MMP12 ligands may be promising. Similar use of other MMP ligands can be considered. Attachment of colloid gold nanoparticles to such ligands would enable selective gold build-up in tumor sites with subsequent enhancement of therapeutic X-ray absorption. The long-term cancer survival rate in the presence of such gold nano-particles during a systemic radiotherapy comprises 86% against only 20% with X-ray alone in mice [30]. The effect arises due to local absorbance and scattering of X-ray energy on the clusters of D-element atoms (gold in this case). In our differential expression studies, some tumor types (glioma, melanoma, small intestine cancer) displayed more tissue-specific over-expression events, while many cancers did not. However, the MMP12 expression profile presented in this work was derived using lung tissue data. This observation makes MMP-based approaches more universal. For more detail see Supporting Materials, Text S1, pp 29–46.

The proposed therapy was described as an example of approaches suggested by the results of our work. Currently, significant investment in time and funds is consumed by the study of molecular signaling associated with cancer targets. At the same time, especially high expression variation associated with such targets questions uniformity of their presence in malignant clonal population and the significance of using blockers against such targets. However, using over-expressing clones as attachment sites for selective delivery of radioactivity appears to be bypassing these difficulties. Absorption and scattering of radiation by such attachment sites would create “killing zones”, encompassing the malignant clones that insufficiently express a particular target and do not depend on it for survival.

### C. The expression variation study and its impact upon identification of successful pharmacological targets

The high cost of new therapeutics against chronic disease drains the resources of society by forcing higher health care spending and by detracting from the vital task of anti-infective development. In the context of SARS outbreak and our new knowledge of pandemic flu genesis it becomes imperative to produce computational signatures: of the anti-infective targets, of the successful targets against chronic disease, and of the ligands capable of being viable leads. Apparently, lagging in these technologies opens vulnerabilities at global scale, considering the issues of microbial drug resistance, bioengineering and bio-terror.

Based on the findings of this report, we rationalized and advanced the criteria of target prioritization, previously published in [31]. In the latter publication we show that the future therapeutic success of a prospective target can be predicted *a-priori* in large integrated datasets, based on the gene's expression behavior. In the current report we attempted to rationalize this link.

Ontological analysis reveals that the genes of high variability class may require more sophisticated orchestration of their functions (P4.13-P4.14). At the same time low variability genes (enzymes, cytoskeleton components, ribosomes) appear to be regulated in a more static (conservative?) manner. Such a result is in agreement with external variability model published in [4]. The functions that require more coordination **are statistically more error-prone** and thus the link between variability and disease can be rationalized at mechanistic level. Namely, the most of variations occur in the expression levels of genes carrying out sophisticated regulatory functions.

As an example, genes expressed only in a particular tissue lineage display more variable expression than the genes expressed systemically (compare Figure 1 and 3). Tissue-specific expression imposes an additional level of control and leads to increased sophistication of transcription regulation. Disease-related gene classes are 4-fold enriched in regulatory functional categories vs. random genes (Figure 7). As compared to random genes, regulators are subject to more sophisticated orchestration due to downstream systemic leverage. The regulators are sought as pharmacological targets, if other criteria are met. Therefore, high variability of a gene's expression may point to the complexity of regulation that is indicative of its signal or adaptive role.

At the same time, broad variations in the levels of the most upstream regulators are unlikely to be compatible with life - therefore distribution of variation across the tiers of significance should be optimized, by maximized population survival. One can observe these principles following the trends discovered in target success analysis (Figures 8.A–C). Indeed, the goal of non-cancer therapy is to normalize the cell population of interest. The effects of such therapy are typically not cytotoxic and thus may be mediated by signal transducers carrying the maximal systemic impact. Such transducers appear to be provided with stricter expression stability controls, and such controls may override the putative increased variability of signal networks. As a result, average variation of successful targets against non-cancer disease (Figure 8.A) is below the one observed for the candidate genes (Figure 4), although also above the random level.

The goal of anti-cancer therapy is to eliminate the cell population of interest and such therapies – in final reckoning – are cytotoxic. Using the uppermost significance tier of signal transducers for such purposes would endanger normal cells. The signal transducers of lower significance tiers may be more important for tumor than norm and a therapeutic window opens. However, at this level of significance the expression stability controls are less strict and the inherent increased expression variability of signal transducers prevails in this tier. As a result, the expression variability of successful anti-cancer targets exceeds the one for candidate genes (Figure 1 and 4). This hypothesis suggests that anti-cancer “targeted” therapy displays fundamental limitation, since the best anti-cancer targets are located in the secondary tier of significance, not in the primary one.

The goal of antibiotic therapy is to eliminate the bacterial population too. Due to wide evolutional (and structural) divergence, there is a “luxury” of inhibiting the most significant tier of functional elements in the prokaryotic cell with minimal risk to normal human tissues. Correspondingly, the prokaryotic targets display increased evolutional conservation and decreased expression variability as compared to random genes (Figure 8.B, C). Instructively, several penicillin-binding proteins exist in *E. coli*, but only dacB (the actual penicillin target) displays low expression variation. The target candidates in development appear to behave similar to random population in this regard and appear to be less evolutionally conserved (Figure 8. B and C ). Our research comprises integrative, multi-facet analysis, and recent years show the progress in this field [32], [33]. Thus, our study identifies additional criteria of optimized antibiotic therapy design and prepares ground for a cost-efficient and rapid development of such a therapy, see Supporting data, Text S1, p 23–24. In this report we did not set a goal of employing all possible classification features and achieving maximal resolution. Rather, it was a demonstration that successful targets in each category display specific trends. In a broader context, this comparative study of therapeutic target variability provided important insight in the limitations of anti-cancer targeted therapy and in the link between disease on-set and variation.

The limited volume of a journal publication does not allow answering all numerous questions raised by our findings. First, there is a paradox: according to Figures 1–7, expression variation does parallel chronic disease. On the other hand, according to the analysis of Figures 8.A–C an anti-variation mechanism appears to protect the most significant tier of biological functions. How a fledging disease overrides such a mechanism of expression stability control?

The answers to this and other questions are provided in Supporting material, Text S1, pp 7–28.

### Supporting Data

The Supporting data are available online at the link: www.mayburd.com

The primary data and processing files in Excel format are designated by letter P. They comprise initial downloads (the folders P1), datasets covering tissue environments in norm (P2 folder “Norm alone”) and in cancer (P2 folder “Cancer alone”). The file P2.4 comprises integrated panel of differential expression. The supporting materials further comprise assembled panel of variability (folders P3.1, P3.2), alignment of expression parameters (including variability) with target mechanistic data (P3.3, P3.4), the files P4 supports ontological analysis. Each Excel file is also described and annotated in its top left part.

Supporting material in Word format is referred to by Text S1 in the text and contains all details not included in the up-front manuscript as well as description of the supporting data.

## Methods

### Datasets and Databases

Large-scale microarray profiling of disease and norm as well as smaller scale datasets were downloaded from Global Expression Omnibus (GEO) platform at NCBI [34]. In particular, Expression Project for Oncology (expO) was downloaded as record GSE2109 at GEO database [35]. The data for normal expression (Human Body Index project) were downloaded as GSE7307 and GSE3526 [34]. Multiple smaller projects describing either cancer expression alone or in comparative norm vs. cancer setting were extracted. In this report U133 Plus 2.0 Affymetrix Array (Santa Clara, CA) was used for all major measurements (see GPL570 platform at GEO for more detail and annotation). Prokaryotic data were derived in Affymetrix GeneChip *E. coli* Antisense Genome Array platform (GPL199, dataset GDS1827).

### Experimental noise reduction

Aggregating of multiple microarray experiments by diverse authors poses unique challenges due to a significant component of technical noise, overlaid with biological variability. Several steps were taken to maximize the benefits of dataset aggregation in terms of signal-to-noise ratio.

#### a) Selection of high quality dataset components of the integrated panel

Low quality datasets were excluded from the analysis if they presented low levels of signal (that may indicate insufficient hybridization to the probes), evidence of missing genes, imputed data, datasets that are too small (<4 samples).

#### b) Minimization of technical variability within a single project component of the panel

The results pertaining to N samples identically processed were defined as “project”. The averages for each sample were computed among 54670 probe-set readings comprising all genes included in U133 Plus 2.0 microarray by Affymetrix (Santa Clara, CA). Each individual gene expression value in the column of 54670 probe-sets was normalized by that average. Variability was measured as a ratio of maximal and minimal outliers in the profile of N normalized samples obtained under identical conditions and representing the same tissue lineage. The ratios (MAX/MIN) were combined in a large-scale panel of 80 values per each gene, each value representing a dataset (project) component of a panel (Supporting materials, folder P3.1.1; Text S1, tables 1–2, page 8).

#### c) Minimization of disproportionate contributions in the integrating panel by “noisy” projects

The MAX/MIN value refers to a project of N samples. MAX/MIN ratios were converted into Z scores:
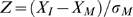
(3)Where X_I_ is the given MAX/MIN value for the i-th probe-set; X_M_ is the average MAX/MIN among 54675 values, σ_M_ is the standard deviation of MAX/MIN among 54675 values (probe-set population of a microarray). X_i_, X_m_ and σ_M_ all refer to ranked values of MAX/MIN. This procedure allows integrating experiments where levels of variability were very different and thus prevents skewing of the resulting panel data in favor of accidentally higher variability values (Supporting materials, folder P3.1.3; Text S1, table 3, page 10)

#### d) Maximization of signal-to-noise ratio by exemption of noise-rich subpopulation

The Z scores were plotted using Q-Q plotting procedure against a theoretical model based on normal distribution [37]. The empirical relative frequencies of high Z score values were compared with the ideal probability values based on the assumption of normal distribution. The concordant regions of Z scores were discarded, since signal-to-noise ratio in such regions is low. The discordant regions of Z scores (on positive side, Z>2) were preserved. Such regions contribute comparatively higher signal-to-noise ratio. The Z scores in the range >2 were summed up and averaged across the panel of 80 expression datasets (P3.1.4; P3.15).

#### e) Minimization of technical noise by comparing large groups of genes

All compared groups and subgroups comprised >150 genes. Finer sub-divisions were avoided.

#### f) Confirmation of trends in related groups

All trends established in this research were confirmed in multiple groups, for example the difference between FDA-approved anti-cancer targets and random genes was supported by the difference between proposed anti-cancer targets and random genes.

### Validation of variability panel data

To exclude a fortuitous panel composition as a source of results, bootstrapping procedure was applied to produce 8 random sub-panels (P3.3). In each sub-panel variability was computed. The procedure produced two sets of 8 values for FDA-approved anti-cancer target variability and random gene variability. The reproducibility in the sets of bootstrap-generated values was assessed by plotting confidence intervals at α  = 0.05.

### Alternative metrics of variability

The metrics comprised: a) coefficient of variation (CV) defined as the ratio of variance in the profile to the average of the profile b) kurtosis (measure of deviation from normal distribution in the profile)

### Expression and differential expression consistency (DEXCON)

To compute gene expression levels, each dataset component of the integrating panel was normalized as described above (each sample divided by array average intensity). The paired panels of 31 matching cancer and normal datasets produced a profile of differential expression values for each probe-set. Those values that exceeded 3-fold up-regulation were preserved and the rest were replaced by zeros, to maximize signal-to-noise ratio. The resulting indexes of consistent up-regulation were computed for the panel of data (P2.3, P2.4).

### Metrics of tissue-specific expression

Microarray data were organized in gene expression panels, each composed of M experiments, each experiment comprising N samples. The expression data were normalized as described above and averaged for each experiment. Thus Normal Expression panel and Cancer Expression panel contained M1 and M2 averaged values each. Several criteria of tissue-specific expression were defined. The MAX_C_ is the maximal expression level in the panel of M2 normalized cancer environments; MAX_N_ is the maximal expression level among M1 normalized disease-free tissue environments, AV is the average level in the norm (average of M1 experiments) and VULNERABLES is the average level measured in the sub-panel of normal tissues most often suffering from side effects of therapy. Cancer expression was characterized by ratios of MAX_C_/MAX_N_; MAX_C_/AV; MAX_C_/VULNERABLES. Simultaneously high ratios indicate a potentially cancer-specific expression level, only minimally expressed in norm. Such profiles were assumed to indicate potential target candidates, specific for a particular cancer lineage and minimally expressed in normal tissues (P5, RIT1).

### Definition of disease-related genes and alignment with expression parameters

The disease-association status follows key-word querying of the database “Genes” at NCBI [38]. The database is filled by text-mining of biomedical literature and comprises all grades of association. No prioritization within the gene list was performed. To produce a query, the most common name of a disease was used, for example “diabetes”, “atherosclerosis”, “aging”, etc. The search results were exported and gene aliases were aligned with the variability, gene expression and DEXCON (P3.2).

### Quantitative ontological analysis

The genes comprising the datasets of study (∼54675 probe-sets) were ranked based on variability and the highest and lowest groups by rank were selected, ∼500 probe-sets in each. The classes were compared by GO-MINER methodology [39], [40]. The statistically representative random group (∼30000 genes, the entire array population) was selected to produce the “total” required by GO-MINER algorithm. The functional enrichment coefficients were computed as ratios:
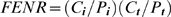
(4)Where *FENR* is functional enrichment coefficient; *C*_i_ is population in the category of interest generated by a studied sub-set of genes; *P*_i_ is population in the studied sub-set of genes; *C*_t_ is population in the same category of interest generated by a total sub-set of genes; *P*_t_ is population in the total subset of genes. The *FENR* for high and low variability groups were compared. The *FENR* were also computed for individual diseases and FDA-approved target datasets. The values of *FENR* were organized in profiles, each functional category corresponding to N values for major human diseases.

To rule out the possibility that any given *FENR* arises accidentally and does not have a biological meaning, 12 randomly selected sets of genes of the size 500–1000 were processed by GO-MINER, establishing a negative control. These values of *FENR* were also organized in profiles per each functional GO-MINER category. The sub-profiles for random genes and diseases were compared using T-test and the resulting p-values were ranked. The most disease-associated functional categories were defined by difference between negative control *FENR* profiles vs. disease-related *FENR* profiles (p<10^−11^). With the T-test p-values being sorted in ascending order, this category forms the top 10% of a rank.

To produce the minimal p-value (the strongest T-test), the disease-related FENR profile has to display minimal scattering, thus the highest ranking belonged to the functional categories corresponding to the most generic features of chronic disease, equally displayed by all pathologies and absent in the negative random control. For illustration see P4, file “Analysis” in SM. The top-ranking and lowest-ranking functional categories, as well as the total list were text-mined with the keywords of interest and the data were plotted.

## Supporting Information

Text S1 Supporing text and in-depth presentation
(0.61 MB DOC)
