# Supplementary material for: Expression Variation: Its Relevance to Emergence of Chronic Disease and to Therapy
Source: PLoS One. 2009 Jun 15;4(6):e5921. doi: 10.1371/journal.pone.0005921 (PMC2692004; doi:10.1371/journal.pone.0005921)
Supplement: Text S1 — Supporing text and in-depth presentation (0.61 MB DOC) [file pone.0005921.s001.doc]

| Supporting Materials  Anatoly Mayburd^1,*^, Irishkan Nogoibaeva^2^ |
| --- |

# Contents

# Introduction ………………………1

# Description of supporting data…………………………………… 1-6

# Questions and answers………. 7-24

# Application of variability studies to optimization of radioisotope therapy (RIT) …………………………………………. .25-41

# INTRODUCTION

The present work addresses a striking fact of increased variability of disease-related gene expression in normal healthy state. Since the same genes may become a part of disease mechanism later in life, the link appears to be causative. In this work we attempted to interpret our data derived on a large-scale experimental material and produce a viable thermodynamics-driven hypothesis connecting these variability-in-the-norm observations and the most general causes of disease. Another aspect are practical applications or our findings. Some practical benefits of this study are immediately apparent and are discussed in the up-front manuscript. Some, however, would become more obvious in the future. In this supplementary material we tried to outline these immediate applications in more detail, see Questions 11 and 12 of the last Q/A section, pages 25-26. We also conducted an elaborate study pointing to the role of expression fluctuations of radioisotope targets in determining cancer survival, pages 24-41 and linked to the theme of expression variation.

# DESCRIPTION OF SUPPORTING DATA

The data are organized in several folders, with the numbering reflecting the progress of the work. The folders are designated by the letter P. They also contain individual files, some of them will be referred by letter P as well. For example P3-P4 means file P4 in the folder P3.

**P1.1 Initial large-scale project data on normal expression (Human Body Index):**

GSE3526_series-1

GSE3526_series-2

GSE7307 Human body index-1

GSE7307 Human body index-2

GSE7307 Human body index-3

**P1.2 Initial large-scale project data on cancer expression (EXPO project):**

GSE2109-1

GSE2109-2

GSE2109-3

GSE2109-4

GSE2109-5

GSE2109-6

GSE2109-7

GSE2109-8

GSE2109-9

**P1.3 Initial gene expression data - small scale projects:**

GDS1249 Dendritic cells

GDS1439 Prostate, benign and cancer

GDS1553 Umbilical vein endothelial cells

GDS1579 Leucocytes generic

GDS1665 Thyroid norm and cancer

GDS1673 Normal lung

GDS1732 Thyroid, normal vs tumor

GDS1973 Prostate normal

GDS2118 CD34+ progenitors from a bone marrow

GDS2216 Monocyte-derived dendritic cells

GDS2221-monocyte derived dendritic cells

GDS1439 Prostate, benign and cancer

GDS2431-CD34+ Hematopoetic progenitor cells, differentiation

GDS2609 Colon mucosa, normal and early cancer

GDS2611 Epidermal keratinocytes

GDS2615 Bronchial mucocilial cells

GDS2628 Bronchial muscle cells

GDS2635 Breast, normal and cancer, micodissected

GDS2697 Normal sperm

GDS2611 Proliferative endometrium normal

GSE2125 Alveolar macrophages

GSE2817 Glyoma

GSE3045 Astrocytes

GSE3141 Primary lung tumors

GSE3325 Prostate, normal, cancer and metastatic

GDS3678 Thyroid norm vs cancer

GSE4452 Multiple myeloma untreated

GSE4587 Normal skin vs melanoma

GSE4757 Brain, normal vs Alzheimer’s

GSE4888 Endometrium normal

GSE4845 melanoma

GSE5060_GPL570 Airway epithelium, smokers vs non_smokers

GSE5079 Astrocytes

GSE5372 Large airway epithelium, pre_ and post_ trauma

GSE5504 Normal monocytes

GSE5850 Normal oocytes

GSE6090 Dendritic cells

GSE6281 Skin

GSE6798 Muscle normal vs disease

GSE6338 Peripheral T_cell lymphoma

GSE8672 Lymphocytes

GSE9452 Colon mucosa normal vs inflamed

GSE9647 huvec endothelial cells

GSE9884 Bone marrow mesenchimal stem cells

GSE6872 Normal sperm

GSE7023 Normal renal and cancer

GSE7476 Bladder norm and cancer

GSE7896 Embryonic stem cells

GSE7846 Endometrium normal and hyperplasia

GSE8302 Normal hepatocytes

GSE8514 Normal adrenal gland

GSE8668 Neutrophils normal

GSE8671 Normal colon mucosa and adenoma

GSE7807 Normal monocytes

**P2.1 Normal expression, individual tissue environments:**

Accumbens

Adipose tissue

Adipose tissue omental

Adrenal gland

Amygdala

Anja cells

Aorta

B-cell resting

Bone marrow

Breast

Bronchus

Caudate

Colon

Coronary artery

Corpus calosum

Dorsal root ganglia

Endometrium

Esophagus

Fallopian tube

GDS1439 Prostate

GDS1673 Normal lung

GDS1973 Prostate normal

GDS2118-CD34+ Progenitors from a bone marrow

GDS2609 Colon mucos, normal vs early cancer

GDS2635 Normal vs cancer, microdissected

Glia, normal vs cancer

GSE3678 Thyroid norm vs cancer

GSE4452 Multiple myeloma untreated

GSE4845 melanoma, norm vs cancer

GDS2615 Bronchial mucocilial cells

GDS3045 Astrocytes

GDS2737 Proliferative endometrium normal

GSE4888 Endometrium normal

GSE5060 Airway epithelium

GSE5372 Large airway epithelium

GSE5850 Normal oocytes

GSE7807 Normal monocytes

GSE7896 Embryonic stem cells

GSE8514 Normal adrenal gland

GSE8668 Neutrophils normal

GSE8671 Normal colon mucosa, adenoma excluded

GSE9894 Bone marrow mesenchimal stem cells

Heart Atrium

Heart Ventricle

Hep G2 cells

Hippocampus

Huvec cell lines

Hypothalamus

Joint tissue sinovium

Kidney

Liver

Liver normal

Lung

Lymph nodes

Medulla

Midbrain

Myometrium

Nipple cross section

Nodose nucleus

Occipital lobe

Oral mucosa

Oral mucosa-1

Ovary

Parietal lobe

Penis normal

Pharyngeal mucosa

Pituitary

Prifrontal cortex

Prostate

Putamen

Salivary gland

Skeletal muscle

Skin

Small intestine

Spinal cord

Spleen

Stomach normal

Substantia nigra

Synovial membrane

Temporal lobe

Testes

Thalamus

Thyroid gland

Tongue normal

Tonsil

Trachea

Trigeminal ganglia

Vagina

Ventral tegmental area

Vestibular nuclei superior

Vulva normal

**P2.2 Cancer expression, individual tissue environments:**

Abdominal wall mass and peritoneum

Appendix area

Bladder

Bone and cartilage

Brain

Breast

Breast-2

Breast-3

Endometrium

Esophagus

Fallopian

GSE2817 Glyoma

GSE4452 Multiple myeloma untreated

GSE4845 Melanoma

Ileum and small intestine

Jejunum

Kidney-2

Kidney-3

Liver cancer

Lung-2

Lung-3

Lung cancer

Lymphatic node

Myometrium cancer

Omentum

Ventral tegmental area

Ovary-2

Omentum

Muscle

Pancreas

Parotid gland

Pelvic mass

Penis

Prostate

Rectosigmoid

Skin

Rectum

Renal pelvis

Retroperitoneal mass and peritoneum

Spleen

Stomach

Testis

Thyroid

Tongue

Ureter

Uterus

Uterus-2

Vulva cancer

**P2.3 Paired datasets, comparing cancer and norm for the same tissue environments:**

Adipose tissue, norm vs cancer

Adrenal gland, norm vs cancer

Bladder, norm vs cancer

Breast, norm vs cancer

Colon, norm vs cancer

Endometrium, normal vs cancer

Esophagus, normal vs cancer

Fallopian tube, normal vs cancer

GDS2609 Colon mucos, normal vs early cancer

GDS2635 Normal vs cancer, microdissected

Glia, normal vs cancer

GSE3678 Thyroid norm vs cancer

GSE4452 Multiple myeloma untreated

GSE4845 melanoma, norm vs cancer</

Ileum and small intestine, norm vs cancer

Kidney, norm vs cancer

Liver, normal vs cancer

Lung, normal vs cancer

Lymphatic node, norm vs cancer, lymphoma

Normal skin vs melanoma

Myometrium, normal vs cancer

Ovary, normal vs.cancer

Pancreas, norm vs cancer

Parotid gland, norm vs cancer

Prostate, norm vs cancer

Small intestine, norm vs cancer

Stomach, normal vs cancer

Thyroid gland, norm vs cancer

Tongue, normal and cancer

Urethra, normal vs cancer

Vagina, normal vs cancer

Vulva, normal vs cancer

**P2.4 Integrated panel of differential expression:**

**P3.1 Large-scale panel of normal variability data:**

P3 Read-me

P3.1.1 Variabilities and expression values, combined panel

P3.1.2 Expression values

P3.1.3 Variabilities, Z scores

P3.1.4 Variabilities, Q-Q plot

P3.1.5 Variabilities, high Z scores only

P3.1.6 Panel averaged variability

**P3.2. The data supporting figures**

P3.2.1 Figure 1

P3.2.2 Figure 2

P3.2.3 Figure 3

P3.2.4 Figure 4

P3.2.5 Figure 5

**P3.3 Bootstrapping subsets**

Subsets 1-8, Analysis

**P4. Ontological analysis of the highest and lowest variability classes:**

P4.1.1 Highest variability genes

P4.1.2 Lowest variability genes

P4.2 Random-1

P4.3 Random-2

P4.4 Random-3

P4.5 Random-4

P4.6 Random-5

P4.7 Random-6

P4.8 Random-7

P4.9 Random-8

P4.10 Random-9

P4.11 Random-10

P4.12 Random-11

P4.13 Aging

P4.14 Alzheimer’s

P4.15 Atherosclerosis

P4.16 Cancer

P4.17 Depression

P4.18 Diabetes

P4.19 Obesity

P4.20 FDA-approved anti-cancer targets

P4.21 FDA-approved non-cancer targets

**P5. Radioisotope therapy (RIT) and variability**

P5.1 RIT1 Original normal and cancer expression

P5.2 RIT2 Target candidates validated by multiple probe-sets

P5.3 RIT3 Expression values for each target were averaged based on multiple probe-sets

P5.4 RIT4 Selection of the best RIT targets

P5.5 RIT5 Annotation of target candidastes

P5.6 RIT6 Glioma targets

# Folders, Brief description

**P1.1 Initial large-scale project data on normal expression (Human Body Index):**

The original data pertaining to a large scale normal expression project can be downloaded at: <http://www.ncbi.nlm.nih.gov/geo/query/acc.cgi?acc=GSE3526>

<http://www.ncbi.nlm.nih.gov/geo/query/acc.cgi?acc=GSE7307> The project GSE3526 is a prototype version of a similarly organized GSE7307. The data are annotated and in case of non-cancer diseases are labeled correspondingly.

**P1.2 Initial large-scale project data on cancer expression (EXPO project):**

The data covering large-scale cancer expression can be found at:

<http://www.ncbi.nlm.nih.gov/geo/query/acc.cgi?acc=GSE2109>

[https://expo.intgen.org](https://expo.intgen.org/)

The projects and individual samples are annotated in detail.

**P1.3 Initial gene expression data - small scale projects:**

This folder contains diverse small-scale projects presenting expression in norm, cancer and paired cancer-norm data, leading to computation of differential expression. Some of the projects were not included in the final panels due to lower quality.

**P2.1 Normal expression, individual tissue environments:**

The data of folders P1.1 and P1.3 were partitioned into individual tissue environments (lung, skin, brain etc.). The datasets originating in P1.1 were designated by the name of the environment “Lung normal” for example. The datasets originating in P1.3 were designated using the dataset numbers and brief descriptions of the content, for example: “GSE8514 Normal adrenal gland”. Each sample in each dataset of P2.1 is named and can be identified using GEO website at NCBI.

**P2.2 Cancer expression, individual tissue environments:**

The data of folders P1.2 and P1.3 were partitioned into individual tissue environments (lung, skin, brain etc.). The datasets originating in P1.2 were designated by the name of the environment “Lung cancer” for example. The datasets originating in P1.3 were designated using the dataset numbers and brief descriptions of the content, for example: “GSE4845 Melanoma”. Each sample in each dataset of P2.2 is named and can be identified using GEO website at NCBI.

**P2.3 Paired datasets, comparing cancer and norm for the same tissue environments:**

After partitioning all data in individual tissue environments, the normal and cancer environments were paired if available. The preference was given to the pairs comprising cancer and norm as a part of original differential expression experiments (folder P1.3).

**P2.4 Integrated panel of differential expression:**

The normal and cancer expression data in P2.3 were normalized by dividing each dataset by its averaged value. Normalized data for norm and for cancer were assembled in a single panel with “norm’ and “cancer” halves. Differential expression was computed for each relevant pair. Also such organization of the panel allows comparing cancer expression in each environment with any combination of normal expression levels, and not only with expression in the matching normal environment. Conversely, environment vs environment were compared and the ratios above the cut-off were awarded the Consistency score of 1. The sum of such scores over a string of N norm-cancer pairs (DEXCON) was accepted as a more objective measure of differential expression than a comparison in a single paired dataset.

**P3.1 Large-scale panel of normal variability data:**

Variabilities in the form of MAX/MIN (maximal outlier vs minimal outlier) were computed for each dataset. Every dataset comprised 54670 genes and for each gene MAX/MIN was computed. The primary ratios were converted into Z scores. The normalized values were integrated into a single large panel of > 80 values. Cut-off criteria were developed by using Q-Q plot comparison of the observed distribution with the normal distribution control. The extreme regions of the empirical distribution (beyond the bulk of population) were deviating from normal distribution. These regions were informative, since they corresponded to the most prominent outliers (Z < -1.5 and Z > 2). Most of random noise, on the contrary, concentrates in the bulk of population (-1.5 < Z < 2). The passing scores filtered by the criteria minimizing the signal-to-noise ratio were accepted in the final panel and summed up.

**P3.2. The data supporting figures**

The folder comprises alignment of expression parameters, established earlier in this project with the disease-association and target data. The results were plotted.

**P3.3 Bootstrapping subsets**

Random compositions of variability panel were produced and variability in random genes and successful anti-cancer target classes were computed and compared in each subset. The nests of variability values for different classes were compared, indicating that the differences between the gene classes do not depend on sub-panel composition.

**P4. Quantitative Ontological analysis**

Each class was represented by multiple (but identical for both) ontological categories and enrichment vs random control was computed. The analysis comprises a comparison of the highest and the lowest variability categories, comparison of random genes and individual diseases, seeking the predominant functional categories characteristic for the major disorders. The ontological analysis further comprises a panel study such that disease relatedness was measured as a p-value of T-test between the sub-profiles of functional enrichment coefficients derived in negative random controls and in grouping of diseases together. The functional categories most generic for the most of chronic diseases were compared with the least related categories. The most and the least related functional categories were compared by using keyword searching.

**P5. Radioisotope therapy (RIT) and variability**

The folder explores application of the current project’s findings to optimization of RIT

# FLOW-CHART OF DATA PROCESSING


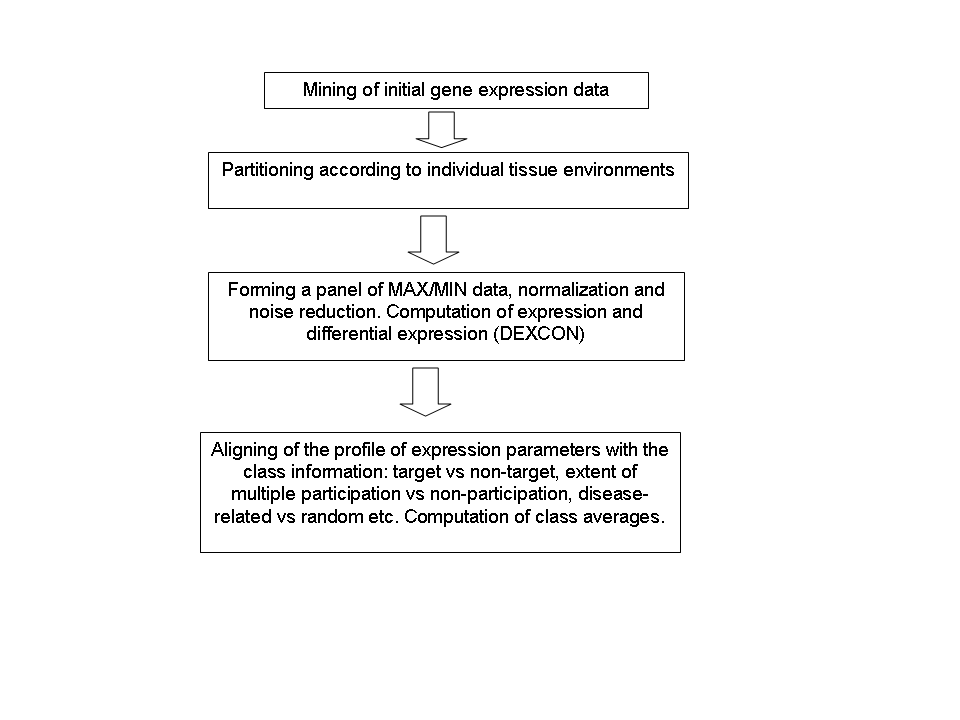


# QUESTIONS AND ANSWERS

**1. What was the methodology of work?**

Answer: In this section we present several methodological aspects of our work in more depth, while the Methods section of the upfront manuscript provides brief discussion of the entire methodology.

**Experimental noise reduction** Aggregating of multiple microarray experiments by diverse authors poses unique challenges due to a significant component of technical noise, overlaid with biological variability. Several steps were taken to maximize the benefits of dataset aggregation in terms of signal-to-noise ratio.

**a) Selection of high quality dataset components of the integrated panel:** Low quality datasets were excluded from the analysis at the outset. Such datasets were defined as presenting low levels of signal (that may indicate insufficient hybridization to the probes), evidence of missing genes, imputed data, datasets that are too small (<4 samples).

**b) Minimization of technical variability within a single project component of the panel:** The results pertaining to N samples identically processed were defined as “project”. After retaining higher quality datasets, the averages for each sample were computed among 54670 probe-set readings comprising all genes included in U133 Plus 2.0 microarray by Affymetrix (Santa Clara, CA). Each individual gene expression value in the column of 54670 probe-sets was normalized by that average. Variability was measured as a ratio of maximal and minimal outliers in the profile of N normalized samples obtained under identical conditions and representing the same tissue lineage. The ratios (MAX/MIN) were combined in a large-scale panel of 80 values per each gene, each value representing a dataset (project) component of a panel. The table below illustrates this design.

Table1. The data lay-out for a single project component of the composite panel.

|  | The profile of N samples, 5 initial are shown. | | | | |
| --- | --- | --- | --- | --- | --- |
|  | Sample 2 | Sample 3 | Sample 4 | Sample 5 …. | Project 5 …. |
| Gene 1 |  |  |  |  |  |
| Gene 2 |  |  |  |  |  |
| Gene 3 |  |  |  |  |  |
| Gene 4 |  |  |  |  |  |
| ……. |  |  |  |  |  |
| Gene 54670 |  |  |  |  |  |

Table 2. The data lay-out for the composite panel comprised of multiple projects. Each project is organized as shown in Table 1.

|  | The profile of projects, 5 initial are shown. | | | | |
| --- | --- | --- | --- | --- | --- |
|  | Project 1 | Project 2 | Project 3 | Project 4 | Project 5 …. |
| Gene 1 |  |  |  |  |  |
| Gene 2 |  |  |  |  |  |
| Gene 3 |  |  |  |  |  |
| Gene 4 |  |  |  |  |  |
| ……. |  |  |  |  |  |
| Gene 54670 |  |  |  |  |  |

**c) Minimization of disproportionate contributions in the integrating panel by “noisy” projects:** The MAX/MIN value refers to a project of N samples. MAX/MIN ratios were converted into Z scores:

$Z=(X_{I} -X_{M} )/\sigma_{M}$ (1)

Where X_I_ is the given MAX/MIN value for the i-th probe-set; X_M_ is the average MAX/MIN among 54675 values, σ_M_ is the standard deviation of MAX/MIN among 54675 values (probe-set population of a microarray). X_i_, X_m_ and σ_M_ all refer to ranked values of MAX/MIN. This procedure allows integrating experiments where levels of variability were very different and thus prevents skewing of the resulting panel data in favor of accidentally higher variability values.

**d) Maximization of signal-to-noise ratio by exemption of noise-rich subpopulation:**

The Z scores were plotted using Q-Q plotting procedure against a theoretical model based on normal distribution [17]. The empirical relative frequencies of high Z score values were compared with the ideal probability values based on the assumption of normal distribution. The concordant regions of Z scores were discarded, since signal-to-noise ratio in such regions is low. The discordant regions of Z scores (on positive side, Z > 2) were preserved. Such regions contribute comparatively higher signal-to-noise ratio. The Z scores in the range > 2 were summed up and averaged across the panel of 80 expression datasets. An alternative way to compute variability was coefficient of variation (CV), obtained as a ratio of standard deviation to the average for the profile of N identically treated samples.

**e) Minimization of technical noise by comparing large groups of genes:** All compared groups and subgroups comprised >150 genes. Finer sub-divisions were avoided.

**f) Confirmation of trends in related groups:** All trends established in this research were confirmed in multiple groups, for example the difference between FDA-approved anti-cancer targets and random genes was supported by the difference between proposed anti-cancer targets and random genes.

**Validation of variability panel data**

To ensure that the differences in variability between disease-related and random genes do not arise due to a fortuitous panel composition, bootstrapping procedure was applied to produce 8 random sub-panels. In each sub-panel variability was computed. The procedure produced two sets of 8 values for FDA-approved anti-cancer target variability and random gene variability. The reproducibility in the sets of bootstrap-generated values was assessed by plotting confidence intervals at α = 0.05.

**Expression and differential expression consistency (DEXCON)**

To compute gene expression levels, each dataset component of the integrating panel was normalized as described above (each sample divided by array average intensity). The paired panels of 31 matching cancer and normal datasets produced a profile of differential expression values for each probe-set. In some samples of these profiles genes were up-regulated in cancer vs. norm and in some were down-regulated. Those values that exceeded 3-fold up-regulation were preserved and the rest were replaced by zeros, to maximize signal-to-noise ratio. The resulting indexes of consistent up-regulation were computed for the panel of data.

Table 3. Illustration of the Z score normalization procedure.

| The direction in which the values are averaged and σ_M_ is computed. |  | The profile of projects, 5 initial are shown.  Z scores are computed for every project | | | | |
| --- | --- | --- | --- | --- | --- | --- |
|  |  | Project 1 | Project 2 | Project 3 | Project 4 | Project 5 …. |
|  | Gene 1 |  |  |  |  |  |
|  | Gene 2 |  |  |  |  |  |
|  | Gene 3 |  |  |  |  |  |
|  | Gene 4 |  |  |  |  |  |
|  | ……. |  |  |  |  |  |
|  | Gene 54670 |  |  |  |  |  |

Table 4. Illustration to computation of DEXCON

The projects are split into pairs. A pair comprises a normal and a cancer dataset, preferably produced in the same experiment. Expression in cancer is compared to expression in norm within a pair. If over-expression is above 2 for cancer vs norm, the DEXCON score receives an increment of 1.

|  | Pairs of Cancer vs Normal expression, ratios Cancer vs Norm | | | | | | | | DEXCON |
| --- | --- | --- | --- | --- | --- | --- | --- | --- | --- |
|  | Pair  1 | Pair  2 | Pair  3 | Pair  4 | Pair 1 | Pair 2 | Pair 3 | Pair 4 |  |
| Gene 1 | 1 | 2 | 1 | 0.5 | 2 | 4 | 1 | 0.5 | 2 |
| Gene 2 | 3 | 0.5 | 2 | 5 | 1.5 | 1 | 3 | 4 | 1 |
| ……. |  |  |  |  |  |  |  |  |  |
| Gene 54670 | 0.5 | 1 | 0.5 | 1.5 | 0.75 | 0.5 | 0.75 | 1 | 0 |

**Metrics of tissue-specific expression**

Microarray data were organized in gene expression panels, each composed of M experiments, each experiment comprising N samples. The expression data were normalized as described above and averaged for each experiment. Thus Normal Expression panel and Cancer Expression panel contained M1 and M2 averaged values each. Experiments correspond to tissue differentiation environments and several criteria of tissue-specific expression were defined. The MAX_C_ is the maximal expression level in the panel of M2 normalized cancer environments; MAX_N_ is the maximal expression level among M1 normalized disease-free tissue environments, AV is the average level in the norm (average of M1 experiments) and VULNERABLES is the average level measured in the sub-panel of normal tissues most often suffering from side effects of therapy. Cancer expression was characterized by ratios of MAX_C_/MAX_N_; MAX_C_/AV; MAX_C_/VULNERABLES. Simultaneously high ratios indicate a potentially cancer-specific expression level, only minimally expressed in norm. Such profiles were assumed to indicate potential target candidates, specific for a particular cancer lineage and minimally expressed in normal tissues.

**Definition of disease-related genes and alignment with expression parameters**

The disease-association status follows key-word querying of the database “Genes” at NCBI [18]. The database is filled by text-mining of biomedical literature and comprises all grades of association. No prioritization within the gene list was performed. To produce a query, the most common name of a disease was used, for example “diabetes”, “atherosclerosis”, “aging”, etc. The search results were exported and gene aliases were aligned with the variability, gene expression and DEXCON.

**Quantitative ontological analysis**

The genes comprising the datasets of study (~54675 probe-sets) were ranked based on variability and the highest and lowest groups by rank were selected, ~ 500 probe-sets in each. The classes were compared by GO-MINER methodology developed by J. Weinstein at NCBI [19]. High-throughput web version was queried. The server classifies the selected gene-sets based on the ontology classification system AMIGO [20]. The statistically representative random group (~ 30000 genes, the entire array population) was selected to produce the “total” required by GO-MINER algorithm.

The functional enrichment coefficients were computed as ratios:

(2)

Where *FENR* is functional enrichment coefficient; *C*_i_ is population in the category of interest generated by a studied sub-set of genes; *P*_i_ is population in the studied sub-set of genes; *C*_t_ is population in the same category of interest generated by a total sub-set of genes; *P*_t_ is population in the total subset of genes. The *FENR* for high and low variability groups were compared. The *FENR* were also computed for individual diseases and FDA-approved target datasets. The values of *FENR* were organized in profiles, each functional category corresponding to N values for major human diseases.

To rule out the possibility that any given *FENR* arises randomly and does not have a biological meaning, 12 randomly selected sets of genes of the size 500-1000 were processed by GO-MINER, using the same AMIGO classification system and establishing a negative control. These values of *FENR* were also organized in profiles per each functional GO-MINER category. The sub-profiles for random genes and diseases were compared using T-test and the resulting p-values were ranked. The most disease-associated functional categories were defined by difference between negative control *FENR* profiles vs. disease-related *FENR* profiles (p < 10^-11^). With the T-test p-values being sorted in ascending order, this category forms the top 10% of a rank.

To produce the minimal p-value (the strongest T-test), the disease-related FENR profile has to display minimal scattering, thus the highest ranking belonged to the functional categories corresponding to the most generic features of chronic

disease, equally displayed by all pathologies and absent in the negative random control.

Table 6. Illustration to the computation of tissue-specific metrics.

MAX C / MAX N – maximal value among cancer expression values is divided by the same on the normal side; MAX C / AVERAGE – maximal value among cancer expression values is divided by average on the normal side; MAX C / VULNERABLES – maximal value among cancer expression values is divided by the average observed in vulnerable tissue lineages (shaded).

|  | Normal expression | | | | Cancer expression | | | | MAX C/ MAX N | MAX C/ AVERAGE | MAX C/ VULNERABLES |
| --- | --- | --- | --- | --- | --- | --- | --- | --- | --- | --- | --- |
|  | Tissue  1 | Tissue  2 | Tissue  3 | Tissue  4 | Tissue  1 | Tissue  2 | Tissue  3 | Tissue  4 |  |  |  |
| Gene 1 | 1 | 2 | 1 | 0.5 | 2 | 4 | 1 | 0.5 | 2 | 3.8 | 2,6 |
| Gene 2 | 3 | 0.5 | 2 | 5 | 1.5 | 1 | 3 | 4 | 0.8 | 2.6 | 2.3 |
| ……. |  |  |  |  |  |  |  |  |  |  |  |
| Gene 54670 | 0.5 | 1 | 0.5 | 1.5 | 0.75 | 0.5 | 0.75 | 1 | 0.6 | 0.25 | 1.33 |

Table 7 Illustration to the quantitative ontological analysis

The genes associated with a disease are classified based on functional categories, 7500 of such categories exist based on AMIGO system.In each category, functional enrichment is computed (FENR), indicating how the selected gene population differs from the total random population. The FENR are organized in profiles, random vs. disease-related. The random subsets are drawn from the total random population, and the size of these subsets equals the size of the disease-related subsets (number of genes involved). These multiple random sub-sets serve the purpose of a negative control to compensate for the combinatorial factors of drawing a finite number of genes out of a greater total population. This negative control and aggregation of multiple values in a panel ensures that the observed trends in FENR are objective. The random and disease-related profiles are compared by T-test and p-values are computed and ranked.

|  | Random 1 | Random 2 | Random 3 | Disease 1 | Disease 2 | Disease 3 | P-value  by T-test |
| --- | --- | --- | --- | --- | --- | --- | --- |
| Category 1 | FENR1 | FENR3 | FENR4 | FENR5 | FENR6 | FENR7 |  |
| Category 2 | FENR1 | FENR3 | FENR4 | FENR5 | FENR6 | FENR7 |  |
| Category 3 | FENR1 | FENR3 | FENR4 | FENR5 | FENR6 | FENR7 |  |
| …… |  |  |  |  |  |  |  |
| Category 7500 | FENR1 | FENR3 | FENR4 | FENR5 | FENR6 | FENR7 |  |

1. **Are these findings valid?**

Answer: The trends presented in this report were established after the interfering factors were neutralized, including technical noise, disproportionate contributions of single dataset components of the panel and accidental observation of the trend due to a fortuitous panel composition. Objectivity of the trend observation was ensured by a large-scale study design, attention to quality of initial data, normalization by converting MAX/MIN ratios into Z scores and thus minimizing the potential disproportionate contributions of outlier datasets in the panel total. The objectivity of the trend was also enhanced by exemption of relatively “noisy” regions of Z-score distribution, concordant with normal distribution model (file P3). Additional reliability was introduced by confirmation of the trend among multiple random vs. disease-related pairs of classes. The trend was also reproducible among random bootstrap sub-panels, excluding fortuitous origin of the result. The quantitative trend in variability re-emerged independently when multiple different target enrichment approaches were applied. The category with the highest proportion of mechanistically relevant targets was displaying the greatest variability. Same applied to the genes known to be associated with multiple diseases as compared to genes associated with just a single disorder or compared with random genes. In all cases, higher variability correlated with the more consistent or prominent participation in diseases.

1. **What is the logical link between expression variability observed in health and disease inception?**

Answer: (a) Health typically pre-dates chronic disease (b) Increased variations of disease-associated genes were measured in health (c) Consequently, variations in disease-related genes precede pathological process (d) Precedence in time is one of the attributes of causality, although is not the only one f) The argument of causality is reinforced by observation (in published literature) of the link between the gene transcription dosage and disease inception: g) The argument of causality is reinforced by the studies pointing to an external component in the mechanism of variability, namely that the regulatory error can propagate from a “player” to a “player”. Thus, the observed variability has homeostatic deregulation as a cause, same as diseases.

1. **Do alternative explanations of this relationship exist?**

Answer: Temporal precedence *per se* cannot imply causality and more work is required to rule out the alternatives. One alternative is that disease-related genes are indeed prone to higher variability, but the latter is not instrumental in the mechanism of disease, being just a “marker” of such genes (hypothesis 2). Increased variability of disease-related genes and chronic diseases may be two sides of a common underlying mechanism, such as a developmental program progressively lessening homeostatic controls in particular regulatory pathways. Action of such a program would be observed as increased variation of expression (A) followed by disease (B). According to this hypothesis, the event B is not caused by A but parallels A. Differentiation between causal and non-causal precedence of A vs B is of principal importance since it defines the paradigm of future approaches to intervention and prophylaxis.

The alternative hypothesis 3 states that high-variability genes are mostly pro-inflammatory and pro-inflammatory gene activation is a well known hallmark of different pathologies. Pro-inflammatory genes may be loosely regulated to provide adaptive diversity and increase the chances of population survival. At the same time, the core mechanism of chronic diseases may not be inflammatory. Thus, the high variability observed in disease-associated subsets may not be causative *per se*, reflecting volatility of the “satellite” inflammatory fraction.

**5. Why MAX/MIN metric was selected?**

Answer: The metric is sensitive and it reflects the presence of outliers. Typically, it is the presence of outliers that points to deregulation. According to these two arguments, MAX/MIN may be the optimal metric. By comparison, Coefficient of Variation is a less sensitive measure.

**6. Why multiple datasets were assem bled in the panel?**

Answer: The objectivity of study is proportional to its size. This effect is achieved not only through increased sample size, but also through increased representation and diversification.

**7. How noisy is this dataset?**

Answer: Choosing the ratio of high-end vs low-end outliers as a metric produces sensitivity, but also increases noise. In addition, the very trend of greater variability in disease-related genes vs. random genes has different strength in different datasets. Overall assessment is that the level of noise in the system is very high and only panel studies of class-averaged values of expression parameters can be meaningful. The work was performed with this understanding as a guiding principle.

1. **How “disease-association” of some genes was defined?**

Answer: Bio-medical literature was mined using text-mining tools at NCBI and the results were integrated in the gene descriptions in NCBI database “Genes”. The bio-medical literature comprised mechanistic studies, knock-out studies, the reports of polymorphisms, mutations, correlations, differential expression and other statistical associations between genes and diseases. These associations are variable in strength, since differential expression or correlative data may only reflect consequences and not causes. By contrast, gene knock-outs may show dramatic impact on emergence or course of disease and display causality. In this report both weakly-linked and strongly-linked sub-categories were NOT differentiated to preserve the size of the classes, considering the level of noise in the system. Such categorization might become a promising route of development for this study in the future. At the same time, FDA-approved drug targets comprise mechanistically relevant category of genes and their display of increased expression variability strengthens their relevance to disease inception. The fact that specific mechanistically relevant genes display the trend of interest (and at maximal level) eliminates the argument that the trend arises due to the noise introduced by inflammatory genes, generically activated in most of pathologies.

**9. Why participation of some genes in multiple diseases is important phenomenon?**

Answer: Participation of ~50% of all disease-related genes in more than one disease points to common principles that guide inception of chronic pathologies. Multiple participations are consistent with the scenario of the same pathways being destabilized in different tissue environments, producing pathologies inherent to given environments, but triggered by similar inception events. The correlation between the extent of multiple participation and variation of gene expression stresses the link between the latter and the most general mechanism of diseases.

**10. Why differential expression and MAX/MIN variation are related?**

Answer: Differential expression is traditionally considered a correlate of participation in a specific disease mechanism. In addition, it may be a manifestation of gene expression instability since alteration of systemic environment in norm vs. cancer would effect practically each and single gene. The same expression instability (deregulation) produces MAX/MIN outlier ratios. Both metrics are inherently related. Thus, differential expression effects of highest magnitude and consistency may carry predominantly mechanistic significance, the rest being just noise of expression deregulation. Indeed, the same genes often demonstrate down regulation and up-regulation in the experiments studying different tissues and sometimes – the same tissue (and treatment) but in different labs. Such inconsistency is unlikely to be explained by a shift of function. More likely the differential expression comprises random deviations from the average that take place during a change of environment.

**11. Is differential expression a valid metric?**

Answer: Differential expression has two components, one is variability-related and the other is mechanism-related. FDA-approved cancer therapy targets display over-expression consistently and are down-regulated more rarely. This predominance of over-expression can be ranked and the highest ranking genes are more likely to be mechanistically involved, as opposed to just reflecting regulatory instability.

**12. What are the implications of our findings for the state of drug development?**

Answer: Our findings may allow development of analytical criteria capable of detecting novel targets of intervention and facilitate development of novel diagnostic procedures. The two-state model adopted in this report assumes destabilized regulatory contours as initial flash-points of disease (see question 14). To exert the effect, such regulatory contours should be characterized by high downstream impact and inadequate feedback from the peer level and upstream regulators. In addition, such contours possess strong autocrine (positive feedback) loops, capable of amplifying the original fluctuation toward dangerous levels. Such positive feedbacks may be direct (literally self-regulation) or indirect, where regulation is mediated by systemic partners of the biological molecule.

Multiple algorithms and computational tools are currently available to detect:

(a) association of genes in pathways

(b) autocrine loops

(c) the extent of downstream impact.

The assessment of non-responsiveness in terms of peer and upstream control is more complicated and more tools have to be developed. One proposal would entail measurement of non-clustering with the bulk of clustering structure. Such non-clustering would correspond to unique regulatory pattern, consistent with inadequate feedback control of the gene by the upstream factors, since most of other profiles appear to respond to these factors and produce large concordant clusters. Uniqueness of regulation as a valid target criterion was demonstrated in prior works and our research provides an additional interpretation why this feature may lead to discovery of efficient drug targets.

The tendency to form autocrine loops may be extracted from the variability profile of a potential target, since initial decline of the gene’s level may lead to additional disproportional decline, while initial increase may lead to additional disproportional increase, according to positive feedback. Thus, variation becomes a primary, but certainly not the only determinant of gene’s role in disease genesis.

Downstream impact can be assessed through measuring of the number of links between the gene of interest and the others, measured as promoter binding events, protein-protein interaction, phosphorylation events. A database of knock-out results, accompanied by microarry and cellomic profiling of each knock out would be invaluable resource in assessment of up-stream and downstream control pattern in genome.

While the concept of disease-producing contour may be ultimately productive and may include a significant proportion of theoretical total target pool, other paradigms of a target may exist as well. Thus, the benefits of using this method toward mining out the most probable target candidate subset appear to be maximal for the diseases relying on positive feedbacks as initiating events.

Also, the method’s indications are unlikely to outweigh the experimental evidence of early target efficiency. Thus, the place of method’s insertion in the target development cycle has to be very early, and for very innovative large scale development programs. The impact of our finding on acceleration of novel drug design is likely to be moderate under the current conditions. Even so, considering the magnitude of investment and narrow profit margins this added efficiency may impact the balance in the pharmaceutical industry.

1. **What are the implications of the method for diagnostics and prognostics field?**

Our results suggest that the ultimate cause of chronic disease is self-perpetuating deregulation of gene functions. Such deregulation may be a consequence of a mutation, but more often than not it appears to emerge from stochastic noise in gene expression. Thus, genetic screening as a concept must be broadened to include not only polymorphisms at the level of gene sequence and splicing, but also the profiles of cell-to-cell variation. Together all three factors would provide reliable correlates regarding regulatory stability in crucial disease-related pathways. Such variability studies would provide the “missing link” making genetic testing complete.

The cell-to-cell variability may be economically measured by novel techniques involving fixing of single cells, their permeabilization, treatment with the plurality of phosphospecific antibodies and analyzing the array of treated cells by high-throughput flow-cytometry. The analytical platforms currently developed by such companies as Perkin-Elmer (and others) allow barcoding and de-convoluting of multiple spectral bands emanated by the nano-dots tagging the antibodies. The specific epitopes comprise not only the phosphorylation sites, but the sites of proteolysis. Cells can be loaded by multiple enzymatic substrates and reactions can be monitored by appearance of specific fluorescence. In other words, a significant fraction of expression levels in genome can be monitored directly in every single cell, and the rest of the level can be deduced computationally, minimizing the gaps. The methodologies of this kind were pioneered by Cellomics (<http://www.cellomics.com/>), and are also known as High Content Analysis.

Assuming that variability profiles are measured, they may provide a second dimension to multivariate predictors correlating the signatures of gene expression with diagnosis, prognosis and predisposition studies. Such signatures are currently used for multiple purposes, however they are not immune against misclassification.

Incorporation of a variability dimension in such predictive tools (at the price of focusing on fewer – but biologically more prominent genes?) may dramatically improve predictive capabilities. The range of questions answerable by such early diagnostic tools may include estimation of risks of developing a particular disease by a particular age, estimation of probability of a disease recurrence, evaluation of overall robustness and longevity, future clinical success of a drug tested in vivo, ability of a given drug to find synergistic partners in other drugs also tested using a similar method. More applications are likely to be produced in the future.

Among such applications may be screening of chemical compounds for the induction of high-longevity variability profiles and subsequent determination of molecular targets of such compounds. Understanding that the topology of regulatory network is probably the most crucial factor in determining life satisfaction, health and longevity, the methods discussed in this article and supplement provide for quantitative metric measuring network stability and correlating with such factors.

Our research thus contributes to the field of personalized prophylactic medicine and would allow FDA assessment and control of marketed goods and diet supplements, claiming the impact on longevity and life satisfaction.

While the impact of individual genes upon the topology of regulatory network is important, its final shape is determined by systemic factors as well. Variability profiles would be capable of providing computational inputs in assessing these systemic forces, while averages measured by current microarray, proteomic and metabolmic methods provide for much inferior metrics. With the average reading being equal, one individual may comprise 10% single cell readings at super-high level and 90% at super-low level. Another individual would demonstrate 50% of moderately affected values in identical cell population. It is apparent that two individuals would differ in regulatory robustness of the particular contour, even if the averaged gene expression levels are identical.

In the process of our studies we noticed that variabilities at different levels do correlate and variations between individuals may correlate with the variation observed between the cells of a single individual. However, such correlation is imperfect and variation between individuals cannot replace measurement of cell-to-cell variations within a single individual.

**14. How homeostatic instability becomes a disease?**

To explain the link between increased variability of disease associated genes and sporadic inception of chronic disease, we proposed a biological network model. The model follows several assumptions, consistent with the current studies:

(1) The biological network is a multioscillator, it includes contours consisting of closely linked activators and repressors.

(2) The genes forming the oscillatory contours (both activators and repressor components) may form positive feedbacks of self-activation and self-repression. Such feedbacks may be either direct or mediated by other systemic factors.

(3) The activators are defined as the components that initiate transcription, translation, metabolite intake and growth while the reverse is true for the repressors.

The general scheme of a regulatory contour is shown below in Figure 1. The constants K_1_, K_2_ (negative feedback constants) and K_3_, K_4_ (positive feedback constants) determine how activators (A) and repressors (R) forming a contour impact themselves and the opposing partners. Each of the constants is the sum of direct (close-range) and long range (system-mediated) impacts of one component upon another, but the latter is negligible. Each constant can be positive, negative or zero. The activities of repressors and activators respectively are linked to growth rate G(t) via the coupling constants K_A_ and K_R_ in the balance equation (3):

G(t) = K_a_ ∑ [A] – K_r_ ∑ [R] (3)

The symbols [A] and [R] may not mean physical concentrations, but reflect the level of activity, the concentrations may decrease at the same or higher level of activity.

Thus, the generic dynamic equations become:

dA/dt = -K_2_ [R] ± K_3_ [A] (4)

dR/dt = K_1_ [A] ± K_4_ [R] (5)

Omitting elementary transformations, the solution of (4)-(5) represents a harmonic oscillator at K_3_ and K_4_= 0, and the solution becomes exponential for ***both*** A and R at K_2_ and K_4_ = 0. For the special case (K_1_>0, K_2_ <0, K_3_ >0, K_4_ = 0), elementary transformations lead to a solution in the form:

A(t)=A_0_exp(ωt) + c (6)

and a quadratic parametric equation:

β^2^-K_3_β+K_1_K_2_ = 0 (7)

The solutions of the parametric equation (7):

β = (K_3_ ± √ (K_3_^2^-4K_1_K_2_))/2 (8)

At K_3_ > 2K_1_K_2_, the discriminant D >0, and the real number solution is exponential

At K_3_ < 2√ K_1_K_2_ , D < 0, and the solution exists in the form of a complex number:

β = (K_3_ ± i√ (4K_1_K_2_ -K_3_^2^))/2 (9)

Substitution of (9) into (6) followed by Euler’s transformation leads to an oscillatory equation:

ΔA(t)=A_0_exp(K_3_/2) (cosωt + *i* sin ωt) (10)

Where ω = √ (4K_1_K_2_ -K_3_^2^) is the effective frequency of the oscillatory process.

The condition K_3_ = 2√ K_1_K_2_ can be interpreted as the bifurcation point when the oscillatory process breaks-out into exponential deregulation. Substitution of (6) in (5) and integration (at K_4_ = 0) gives:

ΔR(t)=(K1/β)ΔA(t) + C_1_ (11)

Similar transformations hold for the case when K_3_ = 0, K_4_ < 0 (a contour with no autocrine self-activation and with autocrine self-repression). Table 1 below reflects the solutions and outcomes for some of the situations. It is apparent that the outcomes of deregulation in different contours are not symmetrical. In case 1, the activity of repressors is linked to that of activators, including transition to exponential break-out state. Combining (11) and (3), obtain:

(12)

Where A(0) and R(0) are the activities of activators and repressors in the pre-fluctuation state.

Assumption of a stationary state and limited growth leads to:

G(0) → 0 (13)

Consequently,

(14)

According to (14), growth begins to deviate from stationary state (that can be one of no growth), when significant activator and repressor levels may both be altered. The impact of these alterations is translated via coupling constants that in turn are determined by tissue differentiation status. In rapidly proliferating tissues activator coupling constant prevails, while in well differentiated tissues repressor coupling constant prevails.

Figure 1. Illustration to the derivation (3)-(14)


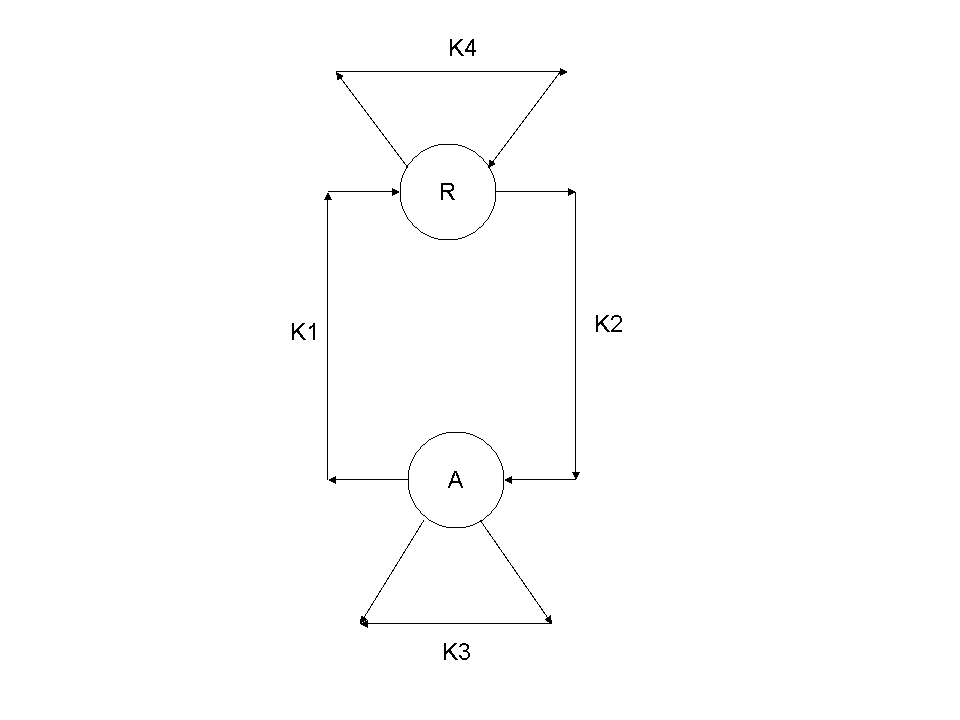


Table 8

Selected oscillatory contour configurations and the consequences of the contour disruption by positive feedbacks. The constants correspond to Figure 6 of main manuscript, positive sign indicating activation and negative sign indicating repression.

| ***Constants*** | ***Solution*** | ***β*** | ***Notes*** |
| --- | --- | --- | --- |
| Case 1: K_3_ >0; K_2_<0; K_1_ >0; K_4_ = 0 | A(t) = A(0)exp(βt) | β = (K_3_ + √ (K_3_^2^-4K_1_K_2_))/2 | Oscillations if K^2^_3_ < 4K_1_K_2_; Exponential break-out if; K^2^_3_ > 4K_1_K_2_ Both activator and repressor components rise exponentially and produce a run-away process if K^2^_3_ > 4K_1_K_2_ |
| Case 2: K_3_ <0; K_2_<0; K_1_ >0; K_4_ = 0 | A(t) = A(0)exp(βt) | β = (-K_3_ - √ (K_3_^2^-4K_1_K_2_))/2 | Oscillations if  K^2^_3_ <4K_1_K_2_;Exponential collapse if; Activator components fall K^2^_3_ > 4K_1_K_2_exponentially and repressor components fall to a new levels sustained through systemic interactions if K^2^_3_ > 4K_1_K_2_ |
| Case 3: K_3_ =0; K_2_<0; K_1_ >0; K_4_ < 0 | R(t) = R(0)exp(βt) | β = (-K_4_ - √ (K_4_^2^-4K_1_K_2_))/2 | Oscillations if K^2^_4_ < 4K_1_K_2_;Exponential collapse of repressors if K^2^_4_ > 4K_1_K_2_ ; Activators rise to new levels based on systemic interactions, may or may not become a part of a run-away process if K^2^_4_ > 4K_1_K_2_ |
| Case 4: K_3_ =0; K_2_<0; K_1_ >0; K_4_ > 0 | R(t) = R(0)exp(βt) | β = (K_4_ + √ (K_4_^2^-4K_1_K_2_))/2 | Oscillations if K^2^_4_ < 4K_1_K_2_;Exponential collapse of activators if K^2^_4_ > 4K_1_K_2_; Repressor components rise exponentially and produce a run-away process if K^2^_4_ > 4K_1_K_2_ |

The analysis above allows the following conclusions:

1) Initially, a homeostatic contour is a stable oscillator

2) A fluctuation activates the positive feedback loop beyond the critical threshold

3) Oscillatory regime switches to exponential

4) In some contours the long-range systemic regulation is insufficient for stabilization

5) Destabilized contour induces destabilization downstream

6) Isolated high-impact contours present the maximal danger of such a scenario and may be sought as therapeutic targets.

References

**[1]** **Colin C. Pritchard, Li Hsu, Jeffrey Delrow, and Peter S. Nelson**

*Project normal: Defining normal variance in mouse gene expression*

PNAS (2001) vol. **98**, no. 23:13266-13271

**[2] Adeline R. Whitney, Maximilian Diehn, Stephen J. Popper, Ash A. Alizadeh, Jennifer C. Boldrick, David A. Relman, and Patrick O. Brown**

*Individuality and variation in gene expression patterns in human blood*

PNAS (2003) vol. **100** , no. 4 :1896-1901

**[3]** Bassett DE, Eisen MB, Boguski MS*. Gene expression informatics—it’s all in your mine*

Nat Genet (1999) **21**:51-55

**[4]** Bar-Even A, Paulsson J, Maheshri N, Carmi M, O’Shea E, Pilpel Y,

Barkai N *Noise in protein expression scales with natural protein abundance*.

Nat Genet (2006) **38**: 636–643

**[5]** Suel GM, Garcia-Ojalvo J, Liberman LM, Elowitz MB *An*

*excitable gene regulatory circuit induces transient cellular differentiation.*

Nature (2006) **440**: 545–550

[6] Simpson, P. *Notch signalling in development: on equivalence groups and asymmetric developmental potential.*

Curr. Opin. Genet. Dev. (1997) **7**: 537–542

**[7] C. Prieto , M.J. Rivas , J.M. Sánchez , J. López-Fidalgo and J. De Las R** *Algorithm to find gene expression profiles of deregulation and identify families of disease-altered genes.*

Bioinformatics (2006) **22**(9):1103-1110

## [8] ****Andrew Singleton, Amanda Myers and John Hardy**** *The law of mass action applied to neurodegenerative disease: a hypothesis concerning the etiology and pathogenesis of complex diseases*

Human Molecular Genetics (2004), **Vol. 13**, Review Issue 1, R123-R126

[9]. Nicole I. Wolf, Erik A. Sistermans, Maria Cundall, Grace M. Hobson, Angelique P. Davis-Williams, Rodger Palmer, Paula Stubbs, Sally Davies, Milda Endziniene, Yvonne Wu, Wui K. Chong, Sue Malcolm, Robert Surtees, James Y. Garbern and Karen J. Woodward *Three or more copies of the proteolipid protein gene PLP1 cause severe Pelizaeus–Merzbacher diseas.*

Brain (2005) **128(4)**:743-751

[10] Magee, J. A., Abdulkadir, S. A. & Milbrandt, J.

Haploinsufficiency at the *Nkx3.1* locus. *A paradigm forstochastic, dosage-sensitive gene regulation during tumor initiation.*

Cancer Cell (2003) **3**, 273–283.

[11] Seidman, J. G. & Seidman, C. *Transcription factor haploinsufficiency: when half a loaf is not enough*. J. Clin. Invest (2002) **109**, 451–455.

[12] Cook, D. L., Gerber, A. N. & Tapscott, S. J. *Modeling stochastic gene expression: implications for haploinsufficiency* (1998)

Proc. Natl Acad. Sci. USA **95**, 15641–15646 .

[13] Lundgren, M. *et al. Transcription factor dosage affects changes in higher order chromatin structure associated with activation of a heterochromatic gene* (2000) Cell **103**, 733–743.

[14] <https://expo.intgen.org/geo/>

[15] <http://www.intgen.org/expo_scientific_release.cfm>

[16]

<http://www.ncbi.nlm.nih.gov/geo/query/acc.cgi?acc=GSE7307>

[17] http://www.itl.nist.gov/div898/handbook/index.htm

[18] http://www.ncbi.nlm.nih.gov/sites/entrez

[19] <http://discover.nci.nih.gov/gominer/htgm.jsp>

[20] <http://www.geneontology.org/amigo/help-front.shtml>

{21] [Rhodes DR](http://www.ncbi.nlm.nih.gov/sites/entrez?Db=pubmed&Cmd=Search&Term="Rhodes DR"%5BAuthor%5D&itool=EntrezSystem2.PEntrez.Pubmed.Pubmed_ResultsPanel.Pubmed_DiscoveryPanel.Pubmed_RVAbstractPlus), [Yu J](http://www.ncbi.nlm.nih.gov/sites/entrez?Db=pubmed&Cmd=Search&Term="Yu J"%5BAuthor%5D&itool=EntrezSystem2.PEntrez.Pubmed.Pubmed_ResultsPanel.Pubmed_DiscoveryPanel.Pubmed_RVAbstractPlus), [Shanker K](http://www.ncbi.nlm.nih.gov/sites/entrez?Db=pubmed&Cmd=Search&Term="Shanker K"%5BAuthor%5D&itool=EntrezSystem2.PEntrez.Pubmed.Pubmed_ResultsPanel.Pubmed_DiscoveryPanel.Pubmed_RVAbstractPlus), [Deshpande N](http://www.ncbi.nlm.nih.gov/sites/entrez?Db=pubmed&Cmd=Search&Term="Deshpande N"%5BAuthor%5D&itool=EntrezSystem2.PEntrez.Pubmed.Pubmed_ResultsPanel.Pubmed_DiscoveryPanel.Pubmed_RVAbstractPlus), [Varambally R](http://www.ncbi.nlm.nih.gov/sites/entrez?Db=pubmed&Cmd=Search&Term="Varambally R"%5BAuthor%5D&itool=EntrezSystem2.PEntrez.Pubmed.Pubmed_ResultsPanel.Pubmed_DiscoveryPanel.Pubmed_RVAbstractPlus), [Ghosh D](http://www.ncbi.nlm.nih.gov/sites/entrez?Db=pubmed&Cmd=Search&Term="Ghosh D"%5BAuthor%5D&itool=EntrezSystem2.PEntrez.Pubmed.Pubmed_ResultsPanel.Pubmed_DiscoveryPanel.Pubmed_RVAbstractPlus), [Barrette T](http://www.ncbi.nlm.nih.gov/sites/entrez?Db=pubmed&Cmd=Search&Term="Barrette T"%5BAuthor%5D&itool=EntrezSystem2.PEntrez.Pubmed.Pubmed_ResultsPanel.Pubmed_DiscoveryPanel.Pubmed_RVAbstractPlus), [Pandey A](http://www.ncbi.nlm.nih.gov/sites/entrez?Db=pubmed&Cmd=Search&Term="Pandey A"%5BAuthor%5D&itool=EntrezSystem2.PEntrez.Pubmed.Pubmed_ResultsPanel.Pubmed_DiscoveryPanel.Pubmed_RVAbstractPlus), [Chinnaiyan AM](http://www.ncbi.nlm.nih.gov/sites/entrez?Db=pubmed&Cmd=Search&Term="Chinnaiyan AM"%5BAuthor%5D&itool=EntrezSystem2.PEntrez.Pubmed.Pubmed_ResultsPanel.Pubmed_DiscoveryPanel.Pubmed_RVAbstractPlus). *Large-scale meta-analysis of cancer microarray data identifies common transcriptional profiles of neoplastic transformation and progression.*

[Proc Natl Acad Sci U S A.](javascript:AL_get(this, 'jour', 'Proc Natl Acad Sci U S A.');)(2004) **101**(25):9309-14. Epub 2004 Jun 7.

[22] **C. J. Zheng, L. Y. Han, C. W. Yap, Z. L. Ji, Z. W. Cao and Y. Z. Chen**

*Therapeutic Targets: Progress of Their Exploration and Investigation of Their Characteristics.* Pharmacol Rev (2006) **58**:259-279.

[23] [Caricasole A](http://www.ncbi.nlm.nih.gov/sites/entrez?Db=pubmed&Cmd=Search&Term="Caricasole A"%5BAuthor%5D&itool=EntrezSystem2.PEntrez.Pubmed.Pubmed_ResultsPanel.Pubmed_DiscoveryPanel.Pubmed_RVAbstractPlus), [Bakker A](http://www.ncbi.nlm.nih.gov/sites/entrez?Db=pubmed&Cmd=Search&Term="Bakker A"%5BAuthor%5D&itool=EntrezSystem2.PEntrez.Pubmed.Pubmed_ResultsPanel.Pubmed_DiscoveryPanel.Pubmed_RVAbstractPlus), [Copani A](http://www.ncbi.nlm.nih.gov/sites/entrez?Db=pubmed&Cmd=Search&Term="Copani A"%5BAuthor%5D&itool=EntrezSystem2.PEntrez.Pubmed.Pubmed_ResultsPanel.Pubmed_DiscoveryPanel.Pubmed_RVAbstractPlus), [Nicoletti F](http://www.ncbi.nlm.nih.gov/sites/entrez?Db=pubmed&Cmd=Search&Term="Nicoletti F"%5BAuthor%5D&itool=EntrezSystem2.PEntrez.Pubmed.Pubmed_ResultsPanel.Pubmed_DiscoveryPanel.Pubmed_RVAbstractPlus), [Gaviraghi G](http://www.ncbi.nlm.nih.gov/sites/entrez?Db=pubmed&Cmd=Search&Term="Gaviraghi G"%5BAuthor%5D&itool=EntrezSystem2.PEntrez.Pubmed.Pubmed_ResultsPanel.Pubmed_DiscoveryPanel.Pubmed_RVAbstractPlus), [Terstappen GC](http://www.ncbi.nlm.nih.gov/sites/entrez?Db=pubmed&Cmd=Search&Term="Terstappen GC"%5BAuthor%5D&itool=EntrezSystem2.PEntrez.Pubmed.Pubmed_ResultsPanel.Pubmed_DiscoveryPanel.Pubmed_RVAbstractPlus).

*Two sides of the same coin: Wnt signaling in neurodegeneration and neuro-oncology.*

[Biosci Rep.](javascript:AL_get(this, 'jour', 'Biosci Rep.');) (2005) **25**(5-6):309-27.

[24] [Farmer A](http://www.ncbi.nlm.nih.gov/sites/entrez?Db=pubmed&Cmd=Search&Term="Farmer A"%5BAuthor%5D&itool=EntrezSystem2.PEntrez.Pubmed.Pubmed_ResultsPanel.Pubmed_DiscoveryPanel.Pubmed_RVAbstractPlus), [Korszun A](http://www.ncbi.nlm.nih.gov/sites/entrez?Db=pubmed&Cmd=Search&Term="Korszun A"%5BAuthor%5D&itool=EntrezSystem2.PEntrez.Pubmed.Pubmed_ResultsPanel.Pubmed_DiscoveryPanel.Pubmed_RVAbstractPlus), [Owen MJ](http://www.ncbi.nlm.nih.gov/sites/entrez?Db=pubmed&Cmd=Search&Term="Owen MJ"%5BAuthor%5D&itool=EntrezSystem2.PEntrez.Pubmed.Pubmed_ResultsPanel.Pubmed_DiscoveryPanel.Pubmed_RVAbstractPlus), [Craddock N](http://www.ncbi.nlm.nih.gov/sites/entrez?Db=pubmed&Cmd=Search&Term="Craddock N"%5BAuthor%5D&itool=EntrezSystem2.PEntrez.Pubmed.Pubmed_ResultsPanel.Pubmed_DiscoveryPanel.Pubmed_RVAbstractPlus), [Jones L](http://www.ncbi.nlm.nih.gov/sites/entrez?Db=pubmed&Cmd=Search&Term="Jones L"%5BAuthor%5D&itool=EntrezSystem2.PEntrez.Pubmed.Pubmed_ResultsPanel.Pubmed_DiscoveryPanel.Pubmed_RVAbstractPlus), [Jones I](http://www.ncbi.nlm.nih.gov/sites/entrez?Db=pubmed&Cmd=Search&Term="Jones I"%5BAuthor%5D&itool=EntrezSystem2.PEntrez.Pubmed.Pubmed_ResultsPanel.Pubmed_DiscoveryPanel.Pubmed_RVAbstractPlus), [Gray J](http://www.ncbi.nlm.nih.gov/sites/entrez?Db=pubmed&Cmd=Search&Term="Gray J"%5BAuthor%5D&itool=EntrezSystem2.PEntrez.Pubmed.Pubmed_ResultsPanel.Pubmed_DiscoveryPanel.Pubmed_RVAbstractPlus), [Williamson RJ](http://www.ncbi.nlm.nih.gov/sites/entrez?Db=pubmed&Cmd=Search&Term="Williamson RJ"%5BAuthor%5D&itool=EntrezSystem2.PEntrez.Pubmed.Pubmed_ResultsPanel.Pubmed_DiscoveryPanel.Pubmed_RVAbstractPlus), [McGuffin P](http://www.ncbi.nlm.nih.gov/sites/entrez?Db=pubmed&Cmd=Search&Term="McGuffin P"%5BAuthor%5D&itool=EntrezSystem2.PEntrez.Pubmed.Pubmed_ResultsPanel.Pubmed_DiscoveryPanel.Pubmed_RVAbstractPlus).

*Medical disorders in people with recurrent depression.*

Br J Psychiatry. (2008) **192**(5):351-5.

[25] [Lechin F](http://www.ncbi.nlm.nih.gov/sites/entrez?Db=pubmed&Cmd=Search&Term="Lechin F"%5BAuthor%5D&itool=EntrezSystem2.PEntrez.Pubmed.Pubmed_ResultsPanel.Pubmed_DiscoveryPanel.Pubmed_RVAbstractPlus), [van der Dijs B](http://www.ncbi.nlm.nih.gov/sites/entrez?Db=pubmed&Cmd=Search&Term="van der Dijs B"%5BAuthor%5D&itool=EntrezSystem2.PEntrez.Pubmed.Pubmed_ResultsPanel.Pubmed_DiscoveryPanel.Pubmed_RVAbstractPlus).

*Central nervous system circuitry involved in the hyperinsulinism syndrome.*

[Neuroendocrinology.](javascript:AL_get(this, 'jour', 'Neuroendocrinology.');) (2006) **84**(4):222-34.

[26] R. Rosmond *Obesity and depression: same disease, different names?* Medical Hypotheses,  **62** , Issue 6 , Pages 976 - 979

[27] [Buckingham JC](http://www.ncbi.nlm.nih.gov/sites/entrez?Db=pubmed&Cmd=Search&Term="Buckingham JC"%5BAuthor%5D&itool=EntrezSystem2.PEntrez.Pubmed.Pubmed_ResultsPanel.Pubmed_DiscoveryPanel.Pubmed_RVAbstractPlus). *Glucocorticoids: exemplars of multi-tasking*

[Br J Pharmacol.](javascript:AL_get(this, 'jour', 'Br J Pharmacol.');) (2006) **147**, 1, S258-68

[28] [Harris RE](http://www.ncbi.nlm.nih.gov/sites/entrez?Db=pubmed&Cmd=Search&Term="Harris RE"%5BAuthor%5D&itool=EntrezSystem2.PEntrez.Pubmed.Pubmed_ResultsPanel.Pubmed_DiscoveryPanel.Pubmed_RVAbstractPlus). *Cyclooxygenase-2 (cox-2) and the inflammogenesis of cancer.* [Subcell Biochem.](javascript:AL_get(this, 'jour', 'Subcell Biochem.');) (2007) **42**:93-126

[29] Gilbert D, Lloyd D. *The living cell: a complex autodynamic multi-oscillator system?*

Cell Biol Int. (2000) **24**(8):569-80.

[30] Hong SH, Ondrey FG, Avis IM, Chen Z, Loukinova E, Cavanaugh PF Jr, Van Waes C, Mulshine JL. (2000) *Cyclooxygenase regulates human oropharyngeal carcinomas via the proinflammatory cytokine IL-6: a general role for inflammation?* FASEB J (2000) **14**(11):1499-507.

[31] **Toby J Gibson** *RuNAway Disease: A two cycle model for transmissible spongiform encephalopathies (TSEs) wherein SINE proliferation drives PrP overproduction*

[Genome Biol.](javascript:AL_get(this, 'jour', 'Genome Biol.');) (2001) **2(7)**:Preprint 0006

[32] Zhao J, Fu Y, Yasvoina M, Shao P, Hitt B, O'Connor T, Logan S, Maus E, Citron M, Berry R, Binder L, Vassar R. *Beta-site amyloid precursor protein cleaving enzyme 1 levels become elevated in neurons around amyloid plaques: implications for Alzheimer's disease pathogenesis.* J Neurosci. (2007) **27(14):**3639-49.

[33] [Croce K](http://www.ncbi.nlm.nih.gov/sites/entrez?Db=pubmed&Cmd=Search&Term="Croce K"%5BAuthor%5D&itool=EntrezSystem2.PEntrez.Pubmed.Pubmed_ResultsPanel.Pubmed_DiscoveryPanel.Pubmed_RVAbstractPlus), [Libby P](http://www.ncbi.nlm.nih.gov/sites/entrez?Db=pubmed&Cmd=Search&Term="Libby P"%5BAuthor%5D&itool=EntrezSystem2.PEntrez.Pubmed.Pubmed_ResultsPanel.Pubmed_DiscoveryPanel.Pubmed_RVAbstractPlus). *Intertwining of thrombosis and inflammation in atherosclerosis*.

[Curr Opin Hematol.](javascript:AL_get(this, 'jour', 'Curr Opin Hematol.');) 2007) **14(1)**:55-61.

[34] [Halaban R](http://www.ncbi.nlm.nih.gov/sites/entrez?Db=pubmed&Cmd=Search&Term="Halaban R"%5BAuthor%5D&itool=EntrezSystem2.PEntrez.Pubmed.Pubmed_ResultsPanel.Pubmed_DiscoveryPanel.Pubmed_RVAbstractPlus), [Fan B](http://www.ncbi.nlm.nih.gov/sites/entrez?Db=pubmed&Cmd=Search&Term="Fan B"%5BAuthor%5D&itool=EntrezSystem2.PEntrez.Pubmed.Pubmed_ResultsPanel.Pubmed_DiscoveryPanel.Pubmed_RVAbstractPlus), [Ahn J](http://www.ncbi.nlm.nih.gov/sites/entrez?Db=pubmed&Cmd=Search&Term="Ahn J"%5BAuthor%5D&itool=EntrezSystem2.PEntrez.Pubmed.Pubmed_ResultsPanel.Pubmed_DiscoveryPanel.Pubmed_RVAbstractPlus), [Funasaka Y](http://www.ncbi.nlm.nih.gov/sites/entrez?Db=pubmed&Cmd=Search&Term="Funasaka Y"%5BAuthor%5D&itool=EntrezSystem2.PEntrez.Pubmed.Pubmed_ResultsPanel.Pubmed_DiscoveryPanel.Pubmed_RVAbstractPlus), [Gitay-Goren H](http://www.ncbi.nlm.nih.gov/sites/entrez?Db=pubmed&Cmd=Search&Term="Gitay-Goren H"%5BAuthor%5D&itool=EntrezSystem2.PEntrez.Pubmed.Pubmed_ResultsPanel.Pubmed_DiscoveryPanel.Pubmed_RVAbstractPlus), [Neufeld G](http://www.ncbi.nlm.nih.gov/sites/entrez?Db=pubmed&Cmd=Search&Term="Neufeld G"%5BAuthor%5D&itool=EntrezSystem2.PEntrez.Pubmed.Pubmed_ResultsPanel.Pubmed_DiscoveryPanel.Pubmed_RVAbstractPlus). *Growth factors, receptor kinases, and protein tyrosine phosphatases in normal and malignant melanocytes.* J Immunother  **(**1992) **12**(3):154-61.

[35] Buee L, Bussiere T, Buee-Scherrer V, Delacourte A and Hof PR *
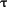
 protein isoforms, phosphorylation and role in neurodegenerative disorders.* Brain Res Rev (2000) **33:** 95-130

[36] **Laurence Canaple, Tomoko Kakizawa and Vincent Laudet** *The Days and Nights of Cancer Cells* Cancer Research (2003)  **63**: 7545-7552

[37] [Bodalina UM](http://www.ncbi.nlm.nih.gov/sites/entrez?Db=pubmed&Cmd=Search&Term="Bodalina UM"%5BAuthor%5D&itool=EntrezSystem2.PEntrez.Pubmed.Pubmed_ResultsPanel.Pubmed_DiscoveryPanel.Pubmed_RVAbstractPlus), [Hammond KD](http://www.ncbi.nlm.nih.gov/sites/entrez?Db=pubmed&Cmd=Search&Term="Hammond KD"%5BAuthor%5D&itool=EntrezSystem2.PEntrez.Pubmed.Pubmed_ResultsPanel.Pubmed_DiscoveryPanel.Pubmed_RVAbstractPlus), [Gilbert DA](http://www.ncbi.nlm.nih.gov/sites/entrez?Db=pubmed&Cmd=Search&Term="Gilbert DA"%5BAuthor%5D&itool=EntrezSystem2.PEntrez.Pubmed.Pubmed_ResultsPanel.Pubmed_DiscoveryPanel.Pubmed_RVAbstractPlus). *Temporal changes in the expression of protein phosphatase 1 and protein phosphatase 2A in proliferating and differentiating murine erythroleukaemia cells.* Cell Biol Int. (2005) **29**(4):287-99.

[38] Mayburd AL, Golovchikova I, Mulshine JL. *Successful anti-cancer drug targets able to pass FDA review demonstrate the identifiable signature distinct from the signatures of random genes and initially proposed targets.* Bioinformatics. (2008) **24**(3):389-95.

[39] **Gennadi V. Glinsky, Takuya Higashiyama and Anna B. Glinskii** *Classification of Human Breast Cancer Using Gene Expression Profiling as a Component of the Survival Predictor Algorithm* Clinical Cancer Research (2004) **Vol. 10**, 2272-2283

[40] **Marja J. Willemse, Taku Seriu, Klaudia Hettinger, Elisabetta d'Aniello, Wim C. J. Hop, E. Renate Panzer-Grümayer, Andrea Biondi, Martin Schrappe, Willem A. Kamps, Guiseppe Masera, Helmut Gadner, Hansjoerg Riehm, Claus R. Bartram, and Jacques J. M. van Dongen** *Detection of minimal residual disease identifies differences in treatment response between T-ALL and precursor B-ALL.* Blood ( 2002) **Vol. 99**, **No. 12**, pp. 4386-4393

[41] **Florence Allantaz, Damien Chaussabel, Dorothee Stichweh, Lynda Bennett, Windy Allman, Asuncion Mejias, Monica Ardura, Wendy Chung, Carol Wise, Karolina Palucka, Octavio Ramilo, Marilynn Punaro, Jacques Banchereau, and Virginia Pascual.**

***Blood leukocyte microarrays to diagnose systemic onset juvenile idiopathic arthritis and follow the response to IL-1 blockade.*** The Journal of Experimental Medicine, **Vol. 204, No. 9,** 2131-2144

[42] [Middleton FA](http://www.ncbi.nlm.nih.gov/sites/entrez?Db=pubmed&Cmd=Search&Term="Middleton FA"%5BAuthor%5D&itool=EntrezSystem2.PEntrez.Pubmed.Pubmed_ResultsPanel.Pubmed_DiscoveryPanel.Pubmed_RVAbstractPlus), [Pato CN](http://www.ncbi.nlm.nih.gov/sites/entrez?Db=pubmed&Cmd=Search&Term="Pato CN"%5BAuthor%5D&itool=EntrezSystem2.PEntrez.Pubmed.Pubmed_ResultsPanel.Pubmed_DiscoveryPanel.Pubmed_RVAbstractPlus), [Gentile KL](http://www.ncbi.nlm.nih.gov/sites/entrez?Db=pubmed&Cmd=Search&Term="Gentile KL"%5BAuthor%5D&itool=EntrezSystem2.PEntrez.Pubmed.Pubmed_ResultsPanel.Pubmed_DiscoveryPanel.Pubmed_RVAbstractPlus), [McGann L](http://www.ncbi.nlm.nih.gov/sites/entrez?Db=pubmed&Cmd=Search&Term="McGann L"%5BAuthor%5D&itool=EntrezSystem2.PEntrez.Pubmed.Pubmed_ResultsPanel.Pubmed_DiscoveryPanel.Pubmed_RVAbstractPlus), [Brown AM](http://www.ncbi.nlm.nih.gov/sites/entrez?Db=pubmed&Cmd=Search&Term="Brown AM"%5BAuthor%5D&itool=EntrezSystem2.PEntrez.Pubmed.Pubmed_ResultsPanel.Pubmed_DiscoveryPanel.Pubmed_RVAbstractPlus), [Trauzzi M](http://www.ncbi.nlm.nih.gov/sites/entrez?Db=pubmed&Cmd=Search&Term="Trauzzi M"%5BAuthor%5D&itool=EntrezSystem2.PEntrez.Pubmed.Pubmed_ResultsPanel.Pubmed_DiscoveryPanel.Pubmed_RVAbstractPlus), [Diab H](http://www.ncbi.nlm.nih.gov/sites/entrez?Db=pubmed&Cmd=Search&Term="Diab H"%5BAuthor%5D&itool=EntrezSystem2.PEntrez.Pubmed.Pubmed_ResultsPanel.Pubmed_DiscoveryPanel.Pubmed_RVAbstractPlus), [Morley CP](http://www.ncbi.nlm.nih.gov/sites/entrez?Db=pubmed&Cmd=Search&Term="Morley CP"%5BAuthor%5D&itool=EntrezSystem2.PEntrez.Pubmed.Pubmed_ResultsPanel.Pubmed_DiscoveryPanel.Pubmed_RVAbstractPlus), [Medeiros H](http://www.ncbi.nlm.nih.gov/sites/entrez?Db=pubmed&Cmd=Search&Term="Medeiros H"%5BAuthor%5D&itool=EntrezSystem2.PEntrez.Pubmed.Pubmed_ResultsPanel.Pubmed_DiscoveryPanel.Pubmed_RVAbstractPlus), [Macedo A](http://www.ncbi.nlm.nih.gov/sites/entrez?Db=pubmed&Cmd=Search&Term="Macedo A"%5BAuthor%5D&itool=EntrezSystem2.PEntrez.Pubmed.Pubmed_ResultsPanel.Pubmed_DiscoveryPanel.Pubmed_RVAbstractPlus), [Azevedo MH](http://www.ncbi.nlm.nih.gov/sites/entrez?Db=pubmed&Cmd=Search&Term="Azevedo MH"%5BAuthor%5D&itool=EntrezSystem2.PEntrez.Pubmed.Pubmed_ResultsPanel.Pubmed_DiscoveryPanel.Pubmed_RVAbstractPlus), [Pato MT](http://www.ncbi.nlm.nih.gov/sites/entrez?Db=pubmed&Cmd=Search&Term="Pato MT"%5BAuthor%5D&itool=EntrezSystem2.PEntrez.Pubmed.Pubmed_ResultsPanel.Pubmed_DiscoveryPanel.Pubmed_RVAbstractPlus). *Gene expression analysis of peripheral blood leukocytes from discordant sib-pairs with schizophrenia and bipolar disorder reveals points of convergence between genetic and functional genomic approaches.* [Am J Med Genet B Neuropsychiatr Genet.](javascript:AL_get(this, 'jour', 'Am J Med Genet B Neuropsychiatr Genet.');) (2005) **136B(1)**:12-25

[43] [Schulz KR](http://www.ncbi.nlm.nih.gov/sites/entrez?Db=pubmed&Cmd=Search&Term="Schulz KR"%5BAuthor%5D&itool=EntrezSystem2.PEntrez.Pubmed.Pubmed_ResultsPanel.Pubmed_DiscoveryPanel.Pubmed_RVAbstractPlus), [Danna EA](http://www.ncbi.nlm.nih.gov/sites/entrez?Db=pubmed&Cmd=Search&Term="Danna EA"%5BAuthor%5D&itool=EntrezSystem2.PEntrez.Pubmed.Pubmed_ResultsPanel.Pubmed_DiscoveryPanel.Pubmed_RVAbstractPlus), [Krutzik PO](http://www.ncbi.nlm.nih.gov/sites/entrez?Db=pubmed&Cmd=Search&Term="Krutzik PO"%5BAuthor%5D&itool=EntrezSystem2.PEntrez.Pubmed.Pubmed_ResultsPanel.Pubmed_DiscoveryPanel.Pubmed_RVAbstractPlus), [Nolan GP](http://www.ncbi.nlm.nih.gov/sites/entrez?Db=pubmed&Cmd=Search&Term="Nolan GP"%5BAuthor%5D&itool=EntrezSystem2.PEntrez.Pubmed.Pubmed_ResultsPanel.Pubmed_DiscoveryPanel.Pubmed_RVAbstractPlus). *Single-cell phospho-protein analysis by flow cytometry*. [Curr Protoc Immunol.](javascript:AL_get(this, 'jour', 'Curr Protoc Immunol.');) (2007) Aug;Chapter **8**:Unit 8.17.

[44] Taylor DL. *Past, present, and future of high content screening and the field of cellomics.* Methods Mol Biol.(2007) **356**:3-18.

**Some practical applications of variability studies: therapeutic exploitation of expression variability and optimization of cancer radioisotope therapy**

**OVERVIEW**

Integrated panel of ~120 Affymetrix U133 platform microarray datasets, comprising 54600 gene probe-sets and ~ 2000 samples was created to represent ~80 of normal and ~ 40 of malignant tissue environments. The genes over-expressed in cancer, but typically non-expressing across all normal environments were identified and validated by internal control and independent (SAGE library) platform, leading to ~ 100 fold differences between a particular cancer and normal average across all tissues. In the context of radiotherapy, such exposure contrasts can in theory overwhelm resistant clonal populations and be curative. Yet most of currently available forms of radioisotope therapies provide either temporary relief or palliative effect. To address these inefficiencies on mechanistic level, variability of expression was compared for RIT candidate targets and random genes. The former displayed 10-fold higher level of variability of expression among individual samples, as compared to random gene expression. Despite differential expression of the averages, certain individual normal samples were over-expressing, creating a potential for side effects. At the same time, certain individual cancer samples were non-expressing, possibly explaining non-responsiveness and recurrence. A practical way of managing this variability was proposed within the frame of personalized medicine, via developing imaging signatures of responding vs. non-responding cases. A choice of isotope may be very significant, according to reliability theory model of therapy outcome.

**INTRODUCTION**

Metastatic cancer is known as the main source of malignancy-related mortality. The reasons behind the fact are diverse. Metastatic cancers are known to be not only disseminated but also resistant to chemotherapy due to continuous process of clonal progression and selection by previous therapies and loss of apoptotic signaling [1,2]. In this regard, radiotherapy acts through more fundamental free-radical mechanism and is less dependent on preservation of apoptotic signaling pathways, making it a more robust approach. At the same time, systemic external beam irradiation may lead to life-threatening side effects, limiting the dosage, even with dose fractionation [3, 4]. Thus, a resistant subset may survive and produce a relapse [4].

In theory, directing a radioactive isotope selectively to cancer cells may be curative. The ideal outcome of such radioisotope therapy is massive exposure of cancer, overwhelming the resistant clone population without prohibitive damage to the norm. However, the practical results are mixed.

Radioisotope therapy of thyroid cancer by I^131^ is one of the success stories of the field. It is known since the 30-s of the 20-th century and it leads up to 45% cure rate, even if the primary tumor has disseminated systemically [5, 6]. Positive response is conditional on preservation of differentiation and a non-responding sub-set exists [7]. Currently, 59 clinical trial studies of radioisotope and radiofrequency ablation therapies are listed by National Institute of Health as recruiting or ongoing [8], the I^131^ based approaches forming a significant proportion. Preferential intake of glucose by most of malignant tumors and of its PET tracer analogue 2-fluoro-2-deoxy-D-glucose (FDG) led to a recently tested “positherapy” [9]. At the doses used, the life span of xenograft-bearing mice was extended, but tumor growth continued. Other examples of targeted RIT comprise [metaiodobenzylguanidine](http://en.wikipedia.org/wiki/Metaiodobenzylguanidine) (MIBG) against [neuroblastoma](http://en.wikipedia.org/wiki/Neuroblastoma) [10], as well as hormone-bound [lutetium](http://en.wikipedia.org/wiki/Lutetium)-177 and [yttrium](http://en.wikipedia.org/wiki/Yttrium)-90 against [neuroendocrine tumors](http://en.wikipedia.org/wiki/Neuroendocrine_tumors) (peptide receptor radionuclide therapy [11]).

The success rate of MIBG is estimated at 35% including complete and partial remission lasting on average for 18 months [12]. The success of Y^90^ peptides was estimated using labeled somatostatin. The complete and partial remission rate in late neuro-endocrine tumors was 28%. [11]. Injection of radioactive glass or resin microspheres into the hepatic artery may be used to radioembolize liver tumors or liver metastases [13], leading to 42.8% response rate (2.1% complete response, 40.7% partial response) according to WHO criteria. There was a biologic tumor response (any decrease in tumor size) of 87%. Overall median survival was 300 days. [Ibritumomab tiuxetan](http://en.wikipedia.org/wiki/Ibritumomab_tiuxetan) (Zevalin), a monoclonal anti-CD20 (MS4A1) antibody conjugated to a molecule of Yttrium-90. as well as [Tositumomab](http://en.wikipedia.org/wiki/Tositumomab) Iodine-131 (Bexxar), a conjugate a of Iodine-131 to the monoclonal antibody anti-CD20 were approved by FDA correspondingly in 2002 and 2003. Long-term responses were seen in 37% (78/211) of patients. A third of these patients had been treated with at least 3 previous therapies, and 37% of them had not responded to their last therapy. The estimated overall survival at 5 years was 53% for all patients treated with 90Y ibritumomab tiuxetan and 81% for long-term responders [14]. In still another approach, incorporation of Strontium-89 in the bones of the patients suffering from metastatic bone pain relieves the symptoms and in some patients leads to complete cessation of pain, but the effect is mostly palliative and not curative. Yet the newest publication presents the data on survival benefits of this therapy [15].

A mixed picture emerges of an approach that is theoretically curative – and yet complete responses practically comprise only ~10% on average across all methodologies. Our analysis attempts to address this under-performance and to suggest ways of improvement.

**METHODS:**

**Datasets and Databases**

The project aggregated large-scale microarray studies of disease and norm as well as smaller scale datasets uploaded to Global Expression Omnibus (GEO) platform at NCBI. [16]. In particular, large scale expression data covering all major human cancers and ~2000 samples were collated within Expression Project for Oncology (expO) and downloaded as record GSE2109 at GEO database [17]. The data for normal expression (Human Body Index project) were downloaded with GSE7307 and GSE3526, comprising another ~1000 samples [18]. Multiple smaller projects describing either cancer expression alone or in comparative norm vs. cancer setting were extracted. The list of these projects is given in Supplementary Materials online. To validate expression data, independent gene expression information was accessed using Serial Analysis Gene Expression platform (SAGE) at Cancer Genome Anatomy Project (CGAP) using Gene Finder [19]. Microarray data by different groups are also available at the same source. In this report U133 Plus 2.0 Affymetrix Array was used exclusively (see GPL570 platform at GEO for more detail and annotation).

**Selection of data**

High-throughput expression data are known for the high level of noise arising due to technical and biological variability, the latter being more important [20]. To ensure that the signal-to-noise ratio is maximized, several levels of noise reduction were incorporated in the data-flow of analysis. Firstly, low quality datasets were excluded from the analysis at the outset. Such datasets were defined as presenting low levels of signal (that may indicate insufficient hybridization to the probes), evidence of missing genes, imputed data, datasets that are too small (<3 samples).

**Normalization**

After retaining higher quality datasets, the averages for each sample were computed among 54670 probe-set readings comprising all genes included in U133 Plus 2.0 Affymetrix microarray (Santa-Clara). Each individual gene expression value in the column of 54670 probe-sets was normalized by that average. The procedure was used in comparing the samples studied in different experiments and by different laboratories.

**Validation of expression levels**

The target candidates represented by multiple Affymetrix probe-sets were sought. Those candidates that show a consistent trend in expression between the probe-sets were selected. The candidates represented by single probe-sets or by insufficiently consistent probe-sets (displaying opposing general trends across probe-sets) were discarded. In addition, the previous study [20] established that agreement between multiple probe-sets representing a gene on Affymetrix platform provides additional information regarding the gene’s cancer affiliation and therapeutic successfulness. Finally, the results of microarray measurements were compared with the results by a completely independent high-throughput expression platform using Serial Analysis Of Gene Expression. The agreeing data were also delimited by the biological function, leaving only those candidates that either correspond to already developed ligands or possess structures conducive to effective ligand development ( “druggable”).

**Variability assessment** (see main manuscript)

**Tissue-specific expression** (see main manuscript)

**RESULTS**

**Tissue-distribution profiles of prospective RIT targets**

Figure 1 presents the profiles of transcript expression levels for several conventional targets in cancer vs. norm setting. It is apparent that the expression levels in some normal tissues often exceed the expression levels in cancers, so that the systemic exposure to the conventional therapeutics becomes significant and may lead to dose-limiting side effects or cancellation of therapy. Mathematically, the relationship between the number N of tumor cells killed per one normal cell (therapeutic index) can be given by the generally known expression:

$N=Exp(\alpha C_{T}-\beta C_{N})$ (1)

Where:

*C_T_, C_N_* are exposures to the therapeutic in tumor and norm correspondingly,  β – are relative sensitivities in tumor and norm correspondingly. Comparing (1) with the tissue distribution profiles of target transcript expression, it is apparent that killing of cancer clone population is incomplete by conventional drugs, especially when the relative sensitivity coefficient decreases for cancer (development of resistance). Conversely, according to the model (1) an agent deleterious to normal population can be well-tolerated if C_T_ >> C_N_. In this report C_N_ can be measured by any of the three parameters, MAX_N_, AV and VULNERABLES, preferably by all three (see definition in the Methods in main manuscript).

. Figure 2 presents the tissue distribution profiles for the target candidates selected based on the criteria outlined above, also see Table 1 below. Comparing and contrasting with the data of Figure 1 points to a much more favorable distribution between norm and cancer for RIT candidate targets, suggested in this report. In most of cases the highest ranking expression in cancer panel (MAX_C_) and normal panel (MAX_N_) both belong to the same tissue environment, pointing to high biological significance of the target expression for this environment.

**Hyper-variability of RIT target expression**

Figures 3A-C present expression data for the transcript levels in prostate, lung and lymphoid tissue correspondingly, metalloproteinase 12 (MMP12) being monitored in lung, olfactory-like receptor OR51E2 being monitored in prostate and CD20 (MS4A1) being monitored in lymphoid tissue. MMP12 and OR51E2 genes encode potentially the best RIT target candidates as shown in Table I and Figure 2, while MS4A1 is an FDA approved target of zevalin. The distributions of the corresponding transcription levels among the norm and tumor biopsies were compared. While the average expression of OR51E2 is significantly higher for cancerous prostate tissue as compared to the average for the norm, the individual samples display a surprising magnitude of variation. In 6 out of 17 normal samples the OR51E2 transcript appears to be present in significant quantities. By contrast in 13 out of 43 tumor samples the same gene does not appear to be significantly expressed. Dramatic variability is also observed for MMP12 lung cancer localization, see Figure 3B. In this case, in only 2 out of 43 normal samples the MMP12 transcript can reach significant levels, comparable or exceeding the same in cancer. Correspondingly, in only 13 out of 83 tumor samples the transcript does not appear to be expressed. The comparative expression data for MS4A1 are presented in Figure 3C. While the target is absent in 4 samples out of 9 for the norm, it is also absent in 1 sample out of 10 for cancer. Such a situation of non-expression in cancer would correspond to insensitivity to therapy. Conversely, in 4 samples in norm and 5 samples in cancer the target is present at comparative levels, indicating likely dose-limiting side effects in the attempt to increase the exposure. When a significant expression in observed in norm, not only relative exposure (cancer vs norm) decreases, but also the absolute amount of the isotope bound to cancer cells, due to redistribution in the normal target expressing tissues..

Figure 4 presents two alternative metrics of expression variability: MAX/MIN and RELVAR. The former metric is the ratio of the maximal to the minimal expression levels in the profile of N samples. RELVAR is relative variation, the ratio of standard deviation to the average level in the profile of N samples. According to Figure 4, MAX/MIN is many fold greater for cancer vs norm for all genes. However, a dramatic difference is observed between MAX/MIN in cancer for RIT candidates (>590) as compared to random genes. (>50). Correspondingly, MAX /MIN for random genes is >38 in cancer and >15 in the norm. While both random genes and RIT target candidates display increased variation in cancer, the latter trend is much more pronounced for the RIT targets. Thus, the magnitude of variation and the presence of cancer vs. norm shift may reveal promising anti-cancer target candidates for the methodologies beyond RIT. The trends observed for MAX/MIN metric were confirmed by a more conservative metric RELVAR, see Figure 4. The data obtained using the smaller variability panel reproduced using a larger-scale version (data not shown).

**Status of exploration and availability of ligands**

The patent publication EP1682113 B1 assigned to GE Healthcare LTD describes use of metalloproteinase MMP ligands as imaging radioisotope conjugates [21]. The ligands of preferential structural class are sulphonamide hydroxamates, and chelator moieties capable of radioisotope ion trapping. The urinary excretion profiles of the conjugates were optimized by the choice of the linker, preferentially polyethylene glycol (PEG). According to our data, MMP11, MMP13, 14, 17 and 19 may also provide favorable exposure contrasts (data not shown). Association of MMP expression with many non-cancer chronic diseases may lead to the presence of the radioligand binding sites in normal but unhealthy tissues. This possibility dictates some caution and necessitates development and deployment of visualization ligands frist, before dose escalation toward the therapeutic range.

Other RIT target candidates included in Table I were also explored. Humanized antibody, HuLuc63 shows therapeutic potential of targeting SLAMF7 in the treatment of multiple myeloma [22]. Since SLAMF7 also shows favorable tissue distribution profile (Table 1 below), conjugating radioactive isotope to HuLuc63 antibody would lead to additional therapeutic effect. The report [23} also describes a monoclonal antibody against REG4 and its tumor growth inhibiting effect. The antibodies to MS4A1 (CD20) exerted anti-lymphoma effect by the mechanism currently under investigation [24], even in the absence of radioactive tag. Internalization of antibodies bound to IGF2 via forming a ternary complex with IGF2 receptor also led to apoptotic cell death [25]. Monoclonal antibodies to FCLRA are described in [26]. Immuno-cytological studies revealed over-expression of CXCL14 at invasion front of pancreatic cancer [27]. Over-expression of chromogranin A (CHGA) in gastric cancers was also established immuno-histologically [28]. Intracellular localization of chromogranin B (CHGB) was studied with immunogold tags in pancreatic neuro-endocrine cells [29]. In 4 cases out of 10 mentioned ligands to RIT candidates, the blockade of such targets led to direct apoptosis or activation of anti-cancer arm of cyto-toxic immune response.

**DISCUSSION**

Expression variability was identified in the past as an essential feature of eukaryotic gene expression and its possible link with disease was highlighted [30]. Yet the magnitude of variability inherent to potential RIT target expression as compared to random genes was an unexpected finding. One result of the study is in observation that the genes most over-expressed in cancer are also hyper-variable in the extent of expression. This hyper-variability appears to correlate with mechanistic roles in the disease (blockade causes anti-tumor effects) and is likely to be fundamentally relevant to the genesis of malignant process. Similar to other attributes of cancer, this property can be both an obstacle to therapy and a source of novel therapeutic developments.

On the obstacle side high variability may lead to non-detection of tumors by visualization protocols, poor therapeutic up-take and absence of cancer-specific ligand binding. On the positive side, high variability allows for the level “spikes” on cancer side and non-expression on normal side developing as random fluctuations. Such fluctuations may theoretically explain a certain percentage of complete long-term responses observed in RIT, as a result of nearly 100% ablation of resistant cancer sub-populations under such favorable conditions. Indeed, referring to expression (1) a fluctuation correction can be introduced:

$N=Exp(\alpha C_{T}\pm\Delta C_{T}-\beta C_{N}\pm\Delta C_{N})$ (2)

If the magnitude of the correction is sufficient and the positive fluctuation on tumor side is matched by the negative fluctuation on the normal side, a favorable profile arises. In this sense MMP12 (Figure 2B, 3B) presents interesting therapeutic opportunities, since it is mostly non-expressing in normal tissues, a number of major malignancies significantly over-expresses it and the ligands are well known, including the imaging probes. The latter category can be easily converted in radio-therapeutics. Other MMPs (see Results) demonstrate similar favorable expression profile, although less “clean” on the normal side.

Other factors, significant to the therapeutic success stem from the analysis of the expression (2). Defining R as the ratio between the target expression level in cancer vs norm, considering material balance and assuming tumor volume (V_t_) insignificant compared to volume of normal compartment (V_n_), one obtains:

$\ln N=(\alpha R\pm\alpha\Delta R-\beta)(D-E)/V_{n}$ (3)

Where D(t) is dosage, E(t) is excretion integral. It is apparent that the “killing zone” determined by effective cross-section of the isotope decay products may effect R. If such a zone extends several millimeters deep relative to the source as is the case with beta-emitters, selectivity may suffer. On the contrary, alpha-emitters would not reach far beyond the cancer cell they bind to and thus their effect is more targeted, improving the parameter R. Recent data considering alpha-emitters appear to be very encouraging [31].

Extreme sensitivity of the therapeutic outcome vs. the parameters of the equation (3) follows the main result of reliability theory:

P(t) = 1 – exp(-λt) (4)

Where P(t) is survival function, λ is the systemic failure coefficient, applicable to the failure of a cancer treatment, interpreted as a multi-component system. This coefficient may be assumed to be proportional to the absolute number of surviving clones.

λ = γ N_R_, (5)

Where N_R_ is the residual tumor burden (occult disease) and γ is the proportionality factor. This analysis presumes that discovery of a single survived clone is an elementary failure event, contributing to the systemic failure rate estimated by λ.

N_R_ = N_tb_/NN_sn_  (6)

P(t) = 0, if NN_sn_ << N_tb_;

P(t) = 1,

if NN_sn_ >> N_tb_ (7)

Where N_sn_ is the accepted number of sacrificed normal cells per a therapy providing given level of N; N_tb_ is tumor burden, absolute number of malignant cells prior to therapy; P(t) is probability of patient’s survival.

It is apparent that a critical value of N exists that (if reached or exceeded) would produce dramatically improved Kaplan-Mayer survival statistics. This parameter N is an exponential function of the subordinate parameters according to equation (3). In turn, the failure rate λ is an exponential function of N, producing a truly leveraged effect of the parameters in equation (3) upon the total outcome.

The targets intended for imaging or RIT may find other promising therapeutic applications beyond systemic radioisotope therapy. They can become the anchors for deposition of pre-targeting nanoparticles, photon activation therapy (PAT) Z-loading agents [32-34], cytotoxic conjugates. For example, recent award-winning proposal at Gotham Prize web-site calls for depth-modulated systemic delivery of kV range X-ray radiation and selective enhancement of its absorbance in tumor regions, by pre-targeting the tumors with gold nanoparticles and other d-elements [35].

An improved therapeutic strategy taking into account expression hyper-variation of targets would be based on a rapid scanning of a number of candidates and selecting the optimal one based on quantitative criteria. Our study indicates that at least 22 potential RIT targets may be available to ensure very significant (20-100 fold) irradiation contrast between norm and cancer. Recent studies confirm significant but moderate correlation between transcription and proteomic data (~0.6, see [36]), thus an additional non-redundant sub-set of RIT candidates may also be discovered to complement the findings of this report. A conservative assessment considering multiple sources of data would lead to an estimate of ~50-100 RIT target candidates per genome, depending on selection stringency. Thus, a number of choices exists ensuring that only the target-ligand pairs with favorable tumor uptake (relative to normal tissues) in quantitative diagnostic radionuclide imaging and stable haematological and biochemical function are considered for therapy [11]. Examination of quantitative imaging features of successful vs. unsuccessful interventions and development of classifiers and cut-offs is another priority on the path of RIT improvement, especially important in light of our findings. Indeed, according to the expressions (3-7), survival is a very sensitive function of cancer vs normal tissue radioligand up-take. The latter is effected by fluctuations. The improvements in image contrast equipment and methodology would allow more precise quantitative assessments, necessitated by highly sensitive N vs R relationship.

**Conclusions**

The factors effecting efficacy of RIT intervention were analyzed. Significantly elevated expression variability of RIT targets vs random genes was discovered in this report. This variability may be the leading obstacle to the success of therapy, but also may create new therapeutic opportunities. A significantly extended diversity of radio-ligands, combined with personalized approach and visualization signatures of efficient response may become a general method of productively exploiting this elevated variability.

Figures

Figure 1

Figure 2

Fig

Figure 3.A

Figure 3.B

Figure 3.C

Figure 4

Table 1.

| **Alias** | **Gene Title**  **and**  **RefSeq Transcript ID** | **Maximal cancer/**  **Maximal norm** | **Maximal cancer / Average norm** | **Maximal cancer/**  **Vulnerable**  **Tissues** | **Localization of maximal expression in cancer** | **Gene Ontology** |
| --- | --- | --- | --- | --- | --- | --- |
|  | **Whole body readiation** | **1** | **1** | **1** |  |  |
| SPON1 | spondin 1, extracellular matrix protein, NM_006108 | 3.80 | 13.40 | 15.02 | Fallopian, Ovary | Extracellular |
| SLITRK6 | SLIT and NTRK-like family, member 6, NM_032229 | 3.26 | 22.58 | 30.73 | Urethra | Cell surface, integral to membrane |
| SLC7A11 | solute carrier family 7, (cationic amino acid transporter, y+ system) member 11, NM_014331 | 2.71 | 11.38 | 21.47 | Urethra | Transporter, ion channel |
| SLC45A2 | solute carrier family 45, member 2,  NM_001012509, NM_016180 | 5.54 | 15.37 | 18.58 | Skin, melanoma | Ion channel |
| SLAMF7 | SLAM family member 7, NM_021181 | 2.52 | 29.48 | 17.96 | Lymphoid | Receptor |
| SLAMF1 | signaling lymphocytic activation molecule family member 1,  NM_003037 | 4.61 | 14.93 | 14.69 | Lymphoid | Receptor |
| REG4 | regenerating islet-derived family, member 4, NM_032044 | 4.01 | 57.77 | 28.94 | Gastro-intestinal | Secreted factor  Sugar binding |
| RARRES1 | retinoic acid receptor responder (tazarotene induced) 1,  NM_002888, NM_206963 | 5.39 | 39.84 | 45.66 | Vagina, Vulva | Cell surface antigen |
| OR51E2 | olfactory receptor, family 51, subfamily E, member 2, NM_030774 | 4.15 | 61.60 | 73.40 | Prostate | Receptor |
| MS4A1 (CD20) | Membrane-spanning 4-domains, subfamily A, member 1,  NM_021950, NM_152866 | 2.01 | 32.28 | 14.85 | Lymphoid | Receptor |
| MMP12 | matrix metallopeptidase 12 (macrophage elastase),  NM_002426 | 7.64 | 105.74 | 57.52 | Reproductive. Gastro-intestinal system, Lung | Secreted |
| MLANA | melan-A, NM_005511 | 8.50 | 89.38 | 140.05 | Skin, melanoma | Integral membrane |
| KRT17 | keratin 17, NM_000422 | 3.08 | 32.30 | 71.64 | tongue | Surface protein |
| IGF2 | insulin-like growth factor 2 (somatomedin A), NM_000612, NM_001007139 | 2.57 | 12.94 | 14.84 | Stomach | Secreted factor |
| FCRLA | Fc receptor-like A, NM_032738 | 4.26 | 25.76 | 18.09 | Lymphoid | Receptor |
| ENPEP | glutamyl aminopeptidase (aminopeptidase A), NM_001977 | 2.14 | 30.95 | 20.25 | Kidney | Cell surface peptidase |
| CXCL14 | chemokine (C-X-C motif) ligand 14, NM_004887 | 2.39 | 11.11 | 8.91 | Kidney | Cytokine |
| CTAG1A | cancer/testis antigen 1B /// cancer/testis antigen 1A, NM_001327, NM_139250 | 3.12 | 18.69 | 15.78 | Melanoma | Surface antigen |
| CLCA2 | chloride channel, calcium activated, family member 2, NM_006536 | 3.17 | 25.58 | 50.97 | Tongue | Ion channel |
| CHGB | chromogranin B (secretogranin 1),  NM_001819 | 2.07 | 23.62 | 104.28 | Adrenal gland, small intestine | Hormone |
| CHGA | chromogranin A (parathyroid secretory protein 1), NM_001275 | 5.77 | 40.76 | 42.93 | Adrenal gland, small intestine | Hormone |

**LEGENGS**

**Figure 1**

Distribution of expression levels across a panel of tissue environments:

Uterus – 1; Cervix -2; Vagina – 3; Vulva – 4; Urethra – 5; Fallopian tubes – 6; Ovary – 7; Breast – 8; Lung – 9; Stomach – 10; Esophagus – 11; Small Intestine – 12; Colon – 13; Glia – 14; Adrenal gland – 15; Salivary gland – 16; Bone Marrow/ Multiple Myeloma – 17; Macrophages/Lymphoma – 18; Skin/Melanoma -19; Liver – 20; Tongue – 21; Thyroid – 22; Kidney – 23; Prostate – 24.

Expression levels in cancer are connected by a dashed line, while the expression levels in norm are connected by a solid line. Expression is dimensionless and is measured as a ratio of the transcript hybridization signal (probe-set average fluorescence reading) to the normalizing sample average, see the Methods section.

**Figure 2**

See the legend to Figure 1.

**Figure 3**

Expression of the prospective RIT target transcript levels in different individuals, norm and cancer. The samples attributable to norm and disease are titled and separated by a vertical solid line. Expression is dimensionless and is determined as described in the legend to Figure 1.

**Figure 4**

Comparative transcript expression variability for anti-cancer targets and random genes.

MAX/MIN indicates the ratio of the highest to the lowest expression levels among M samples obtained and analyzed under identical conditions. RELVAR indicates relative variation of the expression signal (ratio of variation to the average). Both metrics are dimensionless.

Table 1

Parameters of tissue distribution and annotation for the RIT target candidates.

**REFERENCES**

1. Glinsky GV et al. (1997) *Apoptosis and metastasis: increased apoptosis resistance of metastatic cancer cells is associated with the profound deficiency of apoptosis execution mechanisms*. Cancer Lett **115**: 185–193. |

2. [Kajiyama](http://www.ncbi.nlm.nih.gov/pubmed/17611683?ordinalpos=24&itool=EntrezSystem2.PEntrez.Pubmed.Pubmed_ResultsPanel.Pubmed_RVDocSum)  H. et al. (2007)

*Chemoresistance to paclitaxel induces epithelial-mesenchymal transition and enhances metastatic potential for epithelial ovarian carcinoma cells*.

Int J Oncol. **31(2):**277-83.

3.<http://www.bt.cdc.gov/radiation/arsphysicianfactsheet.asp>

4. Rosen E.M. et. al (2000)

*Biological basis or radiation sensitivity.*

Oncology **14**:4

5. Hertz, S. et al. (1938)

*Radioiodine as Indicator in Study of Thyroid Physiology*

Proceedings of the Society of Experimental Biology and Medicine **38**:510-513.

6. Schlumberger, et al. (1996) *Radioactive iodine treatment and external radiotherapy for lung and bone metastases from thyroid carcinoma* J Nucl Med **37**: 598-605.

7. [McIver B,](http://www.ncbi.nlm.nih.gov/pubmed/11742333?ordinalpos=20&itool=EntrezSystem2.PEntrez.Pubmed.Pubmed_ResultsPanel.Pubmed_RVDocSum) et al. (2001)

*Anaplastic thyroid carcinoma: a 50-year experience at a single institution.*

Surgery **130(6):**1028-34.

8. http://www.nci.nih.gov/search/ResultsClinicalTrialsAdvanced.aspx?protocolsearchid=4621175

9. **Renee M. Moadel, Richard H. Weldon, Ellen B. Katz, Ping Lu, Joseph Mani, Mark Stahl, M. Donald Blaufox, Richard G. Pestell, Maureen J. Charron and Ekaterina Dadachova (2005)**

## *Positherapy: Targeted Nuclear Therapy of Breast Cancer with ^18^F-2-Deoxy-2-Fluoro-D-Glucose* **Cancer Research** 65: 698-702

10. <http://acnp.snm.org/index.cfm?PageID=5717&RPID=63>

11. V J Lewington (2003)

*Targeted radionuclide therapy for neuroendocrine tumours*

Endocrine-Related Cancer **10**: 497–501

12. Troncone L, Galli G. (1991) *Proceedings of international workshop on the role of [131I] metaiodobenzylguanidine in the treatment of neural crest tumours*. J Nucl Biol Med **35:** 177-362.

13. [Sato KT](http://www.ncbi.nlm.nih.gov/sites/entrez?Db=pubmed&Cmd=Search&Term="Sato KT"%5BAuthor%5D&itool=EntrezSystem2.PEntrez.Pubmed.Pubmed_ResultsPanel.Pubmed_DiscoveryPanel.Pubmed_RVAbstractPlus), [Lewandowski RJ](http://www.ncbi.nlm.nih.gov/sites/entrez?Db=pubmed&Cmd=Search&Term="Lewandowski RJ"%5BAuthor%5D&itool=EntrezSystem2.PEntrez.Pubmed.Pubmed_ResultsPanel.Pubmed_DiscoveryPanel.Pubmed_RVAbstractPlus), [Mulcahy MF](http://www.ncbi.nlm.nih.gov/sites/entrez?Db=pubmed&Cmd=Search&Term="Mulcahy MF"%5BAuthor%5D&itool=EntrezSystem2.PEntrez.Pubmed.Pubmed_ResultsPanel.Pubmed_DiscoveryPanel.Pubmed_RVAbstractPlus), [Atassi B](http://www.ncbi.nlm.nih.gov/sites/entrez?Db=pubmed&Cmd=Search&Term="Atassi B"%5BAuthor%5D&itool=EntrezSystem2.PEntrez.Pubmed.Pubmed_ResultsPanel.Pubmed_DiscoveryPanel.Pubmed_RVAbstractPlus), [Ryu RK](http://www.ncbi.nlm.nih.gov/sites/entrez?Db=pubmed&Cmd=Search&Term="Ryu RK"%5BAuthor%5D&itool=EntrezSystem2.PEntrez.Pubmed.Pubmed_ResultsPanel.Pubmed_DiscoveryPanel.Pubmed_RVAbstractPlus), [Gates VL](http://www.ncbi.nlm.nih.gov/sites/entrez?Db=pubmed&Cmd=Search&Term="Gates VL"%5BAuthor%5D&itool=EntrezSystem2.PEntrez.Pubmed.Pubmed_ResultsPanel.Pubmed_DiscoveryPanel.Pubmed_RVAbstractPlus), [Nemcek AA Jr](http://www.ncbi.nlm.nih.gov/sites/entrez?Db=pubmed&Cmd=Search&Term="Nemcek AA Jr"%5BAuthor%5D&itool=EntrezSystem2.PEntrez.Pubmed.Pubmed_ResultsPanel.Pubmed_DiscoveryPanel.Pubmed_RVAbstractPlus), [Barakat O](http://www.ncbi.nlm.nih.gov/sites/entrez?Db=pubmed&Cmd=Search&Term="Barakat O"%5BAuthor%5D&itool=EntrezSystem2.PEntrez.Pubmed.Pubmed_ResultsPanel.Pubmed_DiscoveryPanel.Pubmed_RVAbstractPlus), [Benson A 3rd](http://www.ncbi.nlm.nih.gov/sites/entrez?Db=pubmed&Cmd=Search&Term="Benson A 3rd"%5BAuthor%5D&itool=EntrezSystem2.PEntrez.Pubmed.Pubmed_ResultsPanel.Pubmed_DiscoveryPanel.Pubmed_RVAbstractPlus), [Mandal R](http://www.ncbi.nlm.nih.gov/sites/entrez?Db=pubmed&Cmd=Search&Term="Mandal R"%5BAuthor%5D&itool=EntrezSystem2.PEntrez.Pubmed.Pubmed_ResultsPanel.Pubmed_DiscoveryPanel.Pubmed_RVAbstractPlus), [Talamonti M](http://www.ncbi.nlm.nih.gov/sites/entrez?Db=pubmed&Cmd=Search&Term="Talamonti M"%5BAuthor%5D&itool=EntrezSystem2.PEntrez.Pubmed.Pubmed_ResultsPanel.Pubmed_DiscoveryPanel.Pubmed_RVAbstractPlus), [Wong CY](http://www.ncbi.nlm.nih.gov/sites/entrez?Db=pubmed&Cmd=Search&Term="Wong CY"%5BAuthor%5D&itool=EntrezSystem2.PEntrez.Pubmed.Pubmed_ResultsPanel.Pubmed_DiscoveryPanel.Pubmed_RVAbstractPlus), [Miller FH](http://www.ncbi.nlm.nih.gov/sites/entrez?Db=pubmed&Cmd=Search&Term="Miller FH"%5BAuthor%5D&itool=EntrezSystem2.PEntrez.Pubmed.Pubmed_ResultsPanel.Pubmed_DiscoveryPanel.Pubmed_RVAbstractPlus), [Newman SB](http://www.ncbi.nlm.nih.gov/sites/entrez?Db=pubmed&Cmd=Search&Term="Newman SB"%5BAuthor%5D&itool=EntrezSystem2.PEntrez.Pubmed.Pubmed_ResultsPanel.Pubmed_DiscoveryPanel.Pubmed_RVAbstractPlus), [Shaw JM](http://www.ncbi.nlm.nih.gov/sites/entrez?Db=pubmed&Cmd=Search&Term="Shaw JM"%5BAuthor%5D&itool=EntrezSystem2.PEntrez.Pubmed.Pubmed_ResultsPanel.Pubmed_DiscoveryPanel.Pubmed_RVAbstractPlus), [Thurston KG](http://www.ncbi.nlm.nih.gov/sites/entrez?Db=pubmed&Cmd=Search&Term="Thurston KG"%5BAuthor%5D&itool=EntrezSystem2.PEntrez.Pubmed.Pubmed_ResultsPanel.Pubmed_DiscoveryPanel.Pubmed_RVAbstractPlus), [Omary RA](http://www.ncbi.nlm.nih.gov/sites/entrez?Db=pubmed&Cmd=Search&Term="Omary RA"%5BAuthor%5D&itool=EntrezSystem2.PEntrez.Pubmed.Pubmed_ResultsPanel.Pubmed_DiscoveryPanel.Pubmed_RVAbstractPlus), [Salem R](http://www.ncbi.nlm.nih.gov/sites/entrez?Db=pubmed&Cmd=Search&Term="Salem R"%5BAuthor%5D&itool=EntrezSystem2.PEntrez.Pubmed.Pubmed_ResultsPanel.Pubmed_DiscoveryPanel.Pubmed_RVAbstractPlus). (2008) *Unresectable chemorefractory liver metastases: radioembolization with 90Y microspheres--safety, efficacy, and survival.*

Radiology **247(2)**:507-515.

14. [Witzig TE](http://www.ncbi.nlm.nih.gov/sites/entrez?Db=pubmed&Cmd=Search&Term="Witzig TE"%5BAuthor%5D&itool=EntrezSystem2.PEntrez.Pubmed.Pubmed_ResultsPanel.Pubmed_DiscoveryPanel.Pubmed_RVAbstractPlus), [Molina A](http://www.ncbi.nlm.nih.gov/sites/entrez?Db=pubmed&Cmd=Search&Term="Molina A"%5BAuthor%5D&itool=EntrezSystem2.PEntrez.Pubmed.Pubmed_ResultsPanel.Pubmed_DiscoveryPanel.Pubmed_RVAbstractPlus), [Gordon LI](http://www.ncbi.nlm.nih.gov/sites/entrez?Db=pubmed&Cmd=Search&Term="Gordon LI"%5BAuthor%5D&itool=EntrezSystem2.PEntrez.Pubmed.Pubmed_ResultsPanel.Pubmed_DiscoveryPanel.Pubmed_RVAbstractPlus), [Emmanouilides C](http://www.ncbi.nlm.nih.gov/sites/entrez?Db=pubmed&Cmd=Search&Term="Emmanouilides C"%5BAuthor%5D&itool=EntrezSystem2.PEntrez.Pubmed.Pubmed_ResultsPanel.Pubmed_DiscoveryPanel.Pubmed_RVAbstractPlus), [Schilder RJ](http://www.ncbi.nlm.nih.gov/sites/entrez?Db=pubmed&Cmd=Search&Term="Schilder RJ"%5BAuthor%5D&itool=EntrezSystem2.PEntrez.Pubmed.Pubmed_ResultsPanel.Pubmed_DiscoveryPanel.Pubmed_RVAbstractPlus), [Flinn IW](http://www.ncbi.nlm.nih.gov/sites/entrez?Db=pubmed&Cmd=Search&Term="Flinn IW"%5BAuthor%5D&itool=EntrezSystem2.PEntrez.Pubmed.Pubmed_ResultsPanel.Pubmed_DiscoveryPanel.Pubmed_RVAbstractPlus), [Darif M](http://www.ncbi.nlm.nih.gov/sites/entrez?Db=pubmed&Cmd=Search&Term="Darif M"%5BAuthor%5D&itool=EntrezSystem2.PEntrez.Pubmed.Pubmed_ResultsPanel.Pubmed_DiscoveryPanel.Pubmed_RVAbstractPlus), [Macklis R](http://www.ncbi.nlm.nih.gov/sites/entrez?Db=pubmed&Cmd=Search&Term="Macklis R"%5BAuthor%5D&itool=EntrezSystem2.PEntrez.Pubmed.Pubmed_ResultsPanel.Pubmed_DiscoveryPanel.Pubmed_RVAbstractPlus), [Vo K](http://www.ncbi.nlm.nih.gov/sites/entrez?Db=pubmed&Cmd=Search&Term="Vo K"%5BAuthor%5D&itool=EntrezSystem2.PEntrez.Pubmed.Pubmed_ResultsPanel.Pubmed_DiscoveryPanel.Pubmed_RVAbstractPlus), [Wiseman GA](http://www.ncbi.nlm.nih.gov/sites/entrez?Db=pubmed&Cmd=Search&Term="Wiseman GA"%5BAuthor%5D&itool=EntrezSystem2.PEntrez.Pubmed.Pubmed_ResultsPanel.Pubmed_DiscoveryPanel.Pubmed_RVAbstractPlus). (2007) *Long-term responses in patients with recurring or refractory B-cell non-Hodgkin lymphoma treated with yttrium 90 ibritumomab tiuxetan*.

Cancer **109(9)**:1804-10.

15. [Logothetis CJ](http://www.ncbi.nlm.nih.gov/sites/entrez?Db=pubmed&Cmd=Search&Term="Logothetis CJ"%5BAuthor%5D&itool=EntrezSystem2.PEntrez.Pubmed.Pubmed_ResultsPanel.Pubmed_DiscoveryPanel.Pubmed_RVAbstractPlus), [Navone NM](http://www.ncbi.nlm.nih.gov/sites/entrez?Db=pubmed&Cmd=Search&Term="Navone NM"%5BAuthor%5D&itool=EntrezSystem2.PEntrez.Pubmed.Pubmed_ResultsPanel.Pubmed_DiscoveryPanel.Pubmed_RVAbstractPlus), [Lin SH](http://www.ncbi.nlm.nih.gov/sites/entrez?Db=pubmed&Cmd=Search&Term="Lin SH"%5BAuthor%5D&itool=EntrezSystem2.PEntrez.Pubmed.Pubmed_ResultsPanel.Pubmed_DiscoveryPanel.Pubmed_RVAbstractPlus). (2008) *Understanding the biology of bone metastases: key to the effective treatment of prostate cancer.*

[Clin Cancer Res.](javascript:AL_get(this, 'jour', 'Clin Cancer Res.');) **14(6)**:1599-602

16. <http://www.ncbi.nlm.nih.gov/sites/entrez>

17. <https://expo.intgen.org/geo/>

18. <http://www.ncbi.nlm.nih.gov/geo/query/acc.cgi?acc=GSE7307>

.

19. <http://cgap.nci.nih.gov/Genes/>

20. [King C](http://www.ncbi.nlm.nih.gov/sites/entrez?Db=pubmed&Cmd=Search&Term="King C"%5BAuthor%5D&itool=EntrezSystem2.PEntrez.Pubmed.Pubmed_ResultsPanel.Pubmed_DiscoveryPanel.Pubmed_RVAbstractPlus), [Guo N](http://www.ncbi.nlm.nih.gov/sites/entrez?Db=pubmed&Cmd=Search&Term="Guo N"%5BAuthor%5D&itool=EntrezSystem2.PEntrez.Pubmed.Pubmed_ResultsPanel.Pubmed_DiscoveryPanel.Pubmed_RVAbstractPlus), [Frampton GM](http://www.ncbi.nlm.nih.gov/sites/entrez?Db=pubmed&Cmd=Search&Term="Frampton GM"%5BAuthor%5D&itool=EntrezSystem2.PEntrez.Pubmed.Pubmed_ResultsPanel.Pubmed_DiscoveryPanel.Pubmed_RVAbstractPlus), [Gerry NP](http://www.ncbi.nlm.nih.gov/sites/entrez?Db=pubmed&Cmd=Search&Term="Gerry NP"%5BAuthor%5D&itool=EntrezSystem2.PEntrez.Pubmed.Pubmed_ResultsPanel.Pubmed_DiscoveryPanel.Pubmed_RVAbstractPlus), [Lenburg ME](http://www.ncbi.nlm.nih.gov/sites/entrez?Db=pubmed&Cmd=Search&Term="Lenburg ME"%5BAuthor%5D&itool=EntrezSystem2.PEntrez.Pubmed.Pubmed_ResultsPanel.Pubmed_DiscoveryPanel.Pubmed_RVAbstractPlus), [Rosenberg CL](http://www.ncbi.nlm.nih.gov/sites/entrez?Db=pubmed&Cmd=Search&Term="Rosenberg CL"%5BAuthor%5D&itool=EntrezSystem2.PEntrez.Pubmed.Pubmed_ResultsPanel.Pubmed_DiscoveryPanel.Pubmed_RVAbstractPlus). (2005) *Reliability and reproducibility of gene expression measurements using amplified RNA from laser-microdissected primary breast tissue with oligonucleotide arrays.*

[J Mol Diagn.](javascript:AL_get(this, 'jour', 'J Mol Diagn.');) **7**(1):57-64

21. <http://www.epo.org/>

.

22. [Hsi ED](http://www.ncbi.nlm.nih.gov/sites/entrez?Db=pubmed&Cmd=Search&Term="Hsi ED"%5BAuthor%5D&itool=EntrezSystem2.PEntrez.Pubmed.Pubmed_ResultsPanel.Pubmed_DiscoveryPanel.Pubmed_RVAbstractPlus), et al (2008)

*CS1, a Potential New Therapeutic Antibody Target for the Treatment of Multiple Myeloma*

[Clin Cancer Res.](javascript:AL_get(this, 'jour', 'Clin Cancer Res.');) **14**(9):2775-84

23. [Takehara A,](http://www.ncbi.nlm.nih.gov/pubmed/16918991?ordinalpos=7&itool=EntrezSystem2.PEntrez.Pubmed.Pubmed_ResultsPanel.Pubmed_RVDocSum) et al. (2006)

*Novel tumor marker REG4 detected in serum of patients with resectable pancreatic cancer and feasibility for antibody therapy targeting REG4.*

Cancer Sci. **97**(11):1191-7.

24. Uchida J., et. al. (2004)

*The innate mononuclear phagocyte network depletes B lymphocytes through Fc receptor-dependent mechanisms during anti-CD20 antibody immunotherapy.*

[J Exp Med.](javascript:AL_get(this, 'jour', 'J Exp Med.');) **199**(12):1659-69.

25. [Lavrovsky VA](http://www.ncbi.nlm.nih.gov/sites/entrez?Db=pubmed&Cmd=Search&Term="Lavrovsky VA"%5BAuthor%5D&itool=EntrezSystem2.PEntrez.Pubmed.Pubmed_ResultsPanel.Pubmed_DiscoveryPanel.Pubmed_RVAbstractPlus), [Chagin AS](http://www.ncbi.nlm.nih.gov/sites/entrez?Db=pubmed&Cmd=Search&Term="Chagin AS"%5BAuthor%5D&itool=EntrezSystem2.PEntrez.Pubmed.Pubmed_ResultsPanel.Pubmed_DiscoveryPanel.Pubmed_RVAbstractPlus) and [Subkhankulova TN](http://www.ncbi.nlm.nih.gov/sites/entrez?Db=pubmed&Cmd=Search&Term="Subkhankulova TN"%5BAuthor%5D&itool=EntrezSystem2.PEntrez.Pubmed.Pubmed_ResultsPanel.Pubmed_DiscoveryPanel.Pubmed_RVAbstractPlus) (1999)

*Internalization of growth factor-receptor complexes under the influence of antibodies initiates cell apoptosis in vitro.*

[Eur J Cell Biol.](javascript:AL_get(this, 'jour', 'Eur J Cell Biol.');) **78**(3):194-8

26. [Volkova OY,](http://www.ncbi.nlm.nih.gov/pubmed/17451355?ordinalpos=3&itool=EntrezSystem2.PEntrez.Pubmed.Pubmed_ResultsPanel.Pubmed_RVDocSum)  et al. (2007)

*Generation and characterization of monoclonal antibodies specific for human FCRLA*.

Hybridoma (Larchmt).**26**(2):78-85.

.

27. [Wente MN](http://www.ncbi.nlm.nih.gov/sites/entrez?Db=pubmed&Cmd=Search&Term="Wente MN"%5BAuthor%5D&itool=EntrezSystem2.PEntrez.Pubmed.Pubmed_ResultsPanel.Pubmed_DiscoveryPanel.Pubmed_RVAbstractPlus), et al. (2008)

*CXCL14 expression and potential function in pancreatic cancer.*

[Cancer Lett.](javascript:AL_get(this, 'jour', 'Cancer Lett.');) **259**(2):209-17.

28. [Yang S and Chung HC.](http://www.ncbi.nlm.nih.gov/pubmed/18288401?ordinalpos=4&itool=EntrezSystem2.PEntrez.Pubmed.Pubmed_ResultsPanel.Pubmed_RVDocSum) (2008)

*Novel biomarker candidates for gastric cancer.*

Oncol Rep. **19**(3):675-80.

29. [Lukinius A](http://www.ncbi.nlm.nih.gov/sites/entrez?Db=pubmed&Cmd=Search&Term="Lukinius A"%5BAuthor%5D&itool=EntrezSystem2.PEntrez.Pubmed.Pubmed_ResultsPanel.Pubmed_DiscoveryPanel.Pubmed_RVAbstractPlus), [Stridsberg M](http://www.ncbi.nlm.nih.gov/sites/entrez?Db=pubmed&Cmd=Search&Term="Stridsberg M"%5BAuthor%5D&itool=EntrezSystem2.PEntrez.Pubmed.Pubmed_ResultsPanel.Pubmed_DiscoveryPanel.Pubmed_RVAbstractPlus), [Wilander E](http://www.ncbi.nlm.nih.gov/sites/entrez?Db=pubmed&Cmd=Search&Term="Wilander E"%5BAuthor%5D&itool=EntrezSystem2.PEntrez.Pubmed.Pubmed_ResultsPanel.Pubmed_DiscoveryPanel.Pubmed_RVAbstractPlus) (2003)

*Cellular expression and specific intragranular localization of chromogranin A, chromogranin B, and synaptophysin during ontogeny of pancreatic islet cells: an ultrastructural study*

[Pancreas.](javascript:AL_get(this, 'jour', 'Pancreas.');) 27(1):38-46.

## **30.** ****Andrew Singleton, Amanda Myers and John Hardy (2004)**** *The law of mass action applied to neurodegenerative disease: a hypothesis concerning the etiology and pathogenesis of complex diseases*. Human Molecular Genetics, Vol. 13, Review Issue 1, R123-R126

31. [Dahle J](http://www.ncbi.nlm.nih.gov/sites/entrez?Db=pubmed&Cmd=Search&Term="Dahle J"%5BAuthor%5D&itool=EntrezSystem2.PEntrez.Pubmed.Pubmed_ResultsPanel.Pubmed_DiscoveryPanel.Pubmed_RVAbstractPlus), [Borrebaek J](http://www.ncbi.nlm.nih.gov/sites/entrez?Db=pubmed&Cmd=Search&Term="Borrebaek J"%5BAuthor%5D&itool=EntrezSystem2.PEntrez.Pubmed.Pubmed_ResultsPanel.Pubmed_DiscoveryPanel.Pubmed_RVAbstractPlus), [Jonasdottir TJ](http://www.ncbi.nlm.nih.gov/sites/entrez?Db=pubmed&Cmd=Search&Term="Jonasdottir TJ"%5BAuthor%5D&itool=EntrezSystem2.PEntrez.Pubmed.Pubmed_ResultsPanel.Pubmed_DiscoveryPanel.Pubmed_RVAbstractPlus), [Hjelmerud AK](http://www.ncbi.nlm.nih.gov/sites/entrez?Db=pubmed&Cmd=Search&Term="Hjelmerud AK"%5BAuthor%5D&itool=EntrezSystem2.PEntrez.Pubmed.Pubmed_ResultsPanel.Pubmed_DiscoveryPanel.Pubmed_RVAbstractPlus), [Melhus KB](http://www.ncbi.nlm.nih.gov/sites/entrez?Db=pubmed&Cmd=Search&Term="Melhus KB"%5BAuthor%5D&itool=EntrezSystem2.PEntrez.Pubmed.Pubmed_ResultsPanel.Pubmed_DiscoveryPanel.Pubmed_RVAbstractPlus), [Bruland ØS](http://www.ncbi.nlm.nih.gov/sites/entrez?Db=pubmed&Cmd=Search&Term="Bruland ØS"%5BAuthor%5D&itool=EntrezSystem2.PEntrez.Pubmed.Pubmed_ResultsPanel.Pubmed_DiscoveryPanel.Pubmed_RVAbstractPlus), [Press OW](http://www.ncbi.nlm.nih.gov/sites/entrez?Db=pubmed&Cmd=Search&Term="Press OW"%5BAuthor%5D&itool=EntrezSystem2.PEntrez.Pubmed.Pubmed_ResultsPanel.Pubmed_DiscoveryPanel.Pubmed_RVAbstractPlus), [Larsen RH](http://www.ncbi.nlm.nih.gov/sites/entrez?Db=pubmed&Cmd=Search&Term="Larsen RH"%5BAuthor%5D&itool=EntrezSystem2.PEntrez.Pubmed.Pubmed_ResultsPanel.Pubmed_DiscoveryPanel.Pubmed_RVAbstractPlus). (2007) *Targeted cancer therapy with a novel low-dose rate alpha-emitting radioimmunoconjugate.* [Blood.](javascript:AL_get(this, 'jour', 'Blood.');) **110(6)**:2049-56.

32. Aziz EF et al. (2006) *Novel approach in radionuclide tumor therapy: dose enhancement by high-Z element contrast agents*. Cancer Biother Radiopharm. **21(3):**181-93.

33. Hainfeld JF et al. (2004) *The use of gold nanoparticles to enhance radiotherapy in mice.*

Phys Med Biol. **49(18)**:N309-15.

34. Verhaegen F et al. (2005) *Dosimetric and microdosimetric study of contrast-enhanced*

*radiotherapy with kilovolt x-rays*. Phys Med Biol. **50(15)**:3555-69.

35. <http://www.gothamprize.org/files/public/idea34.pdf>

36. Michael W Schmidt, Andres Houseman, Alexander R Ivanov, and Dieter A Wolf (2007)

*Comparative proteomic and transcriptomic profiling of the fission yeast Schizosaccharomyces pombe*

Mol Systems Biology **3**: 79.
